# Generation of antibodies to an extracellular region of the transporters Glut1/Glut4 by immunization with a designed antigen

**DOI:** 10.1016/j.jbc.2024.105640

**Published:** 2024-01-09

**Authors:** Taichi Sumikawa, Makoto Nakakido, Ryo Matsunaga, Daisuke Kuroda, Satoru Nagatoishi, Kouhei Tsumoto

**Affiliations:** 1 Department of Bioengineering, School of Engineering, The University of Tokyo, Tokyo, Japan; 2 Department of Chemistry and Biotechnology, School of Engineering, The University of Tokyo, Tokyo, Japan; 3 Research Center for Drug and Vaccine Development, National Institute of Infectious Diseases, Tokyo, Japan; 4 Laboratory of Medical Proteomics, The Institute of Medical Science, The University of Tokyo, Tokyo, Japan

**Keywords:** generation of antibodies, protein engineering, secondary structure, membrane protein, molecular dynamics simulation

## Abstract

Monoclonal antibodies are one of the fastest growing class of drugs. Nevertheless, relatively few biologics target multispanning membrane proteins because of technical challenges. To target relatively small extracellular regions of multiple membrane-spanning proteins, synthetic peptides, which are composed of amino acids corresponding to an extracellular region of a membrane protein, are often utilized in antibody discovery. However, antibodies to these peptides often do not recognize parental membrane proteins. In this study, we designed fusion proteins in which an extracellular helix of the membrane protein glucose transporter 1 (Glut1) was grafted onto the scaffold protein Adhiron. In the initial design, the grafted fragment did not form a helical conformation. Molecular dynamics simulations of full-length Glut1 suggested the importance of intramolecular interactions formed by surrounding residues in the formation of the helical conformation. A fusion protein designed to maintain such intramolecular interactions did form the desired helical conformation in the grafted region. We then immunized an alpaca with the designed fusion protein and obtained VHH (variable region of heavy-chain antibodies) using the phage display method. The binding of these VHH antibodies to the recombinant Glut1 protein was evaluated by surface plasmon resonance, and their binding to Glut1 on the cell membrane was further validated by flow cytometry. Furthermore, we also succeeded in the generation of a VHH against another integral membrane protein, glucose transporter 4 (Glut4) with the same strategy. These illustrates that our combined biochemical and computational approach can be applied to designing other novel fusion proteins for generating site-specific antibodies.

Monoclonal antibodies (mAbs) as therapeutics are the fastest growing class of drugs on the market (1). Their high level of specificity and affinity to diverse target molecules results in a high level of efficacy and fewer adverse events than other therapies. Furthermore, mAbs can be applied to a wide range of therapeutic targets because of their modes of action (2).

Most antibody therapeutics developed to date have targeted soluble mediators and cell surface receptors that consist of a large extracellular domain with a single transmembrane region (3). In addition to these target proteins, a significant opportunity exists to exploit antibodies to target more complex integral membrane proteins such as G protein–coupled receptors or ion channels, which are associated with a variety of pathologies (4, 5). However, relatively few biologics targeting multispanning membrane proteins have been marketed to date. This reflects the technical challenges involved in isolating mAbs against the extracellular loops of these proteins. It is difficult to prepare multispanning membrane proteins as recombinant proteins (6), and the extracellular loops of these membrane proteins are typically small and thus difficult for antibodies to recognize. Although synthetic peptides, which are composed of amino acids corresponding to an extracellular region of a membrane protein, are often utilized in antibody discovery, it is difficult for linear peptides to maintain the conformational features of native membrane proteins, and therefore, antibodies to these peptides often do not recognize parental membrane proteins.

The variable region of heavy-chain antibodies, referred to as VHH or Nanobody, is a functional single domain protein with favorable characteristics, such as high solubility and stability and high expression level in *Escherichia coli* expression systems (7, 8). While the affinity and specificity toward antigens are comparable to those of conventional antibodies, VHHs tend to have an epitope shape distinct from that of immunoglobulin G (IgG) antibodies (9, 10). Given that the size of the paratope of VHHs, which consists of three complementarity-determining region (CDR) loops, is smaller than that of conventional antibodies, VHHs may have an advantage in targeting a limited extracellular region of multiple membrane-spanning proteins.

Glucose transporter 1 (Glut1) is a membrane protein involved in the transport of glucose across the cell membrane (11). As is the case for many multiple membrane-spanning proteins, such as G protein–coupled receptors, analysis of the crystal structure of Glut1 revealed that the cell surface–exposed extracellular region is limited (12). In contrast, Glut1 has a large intracellular region composed of an intracellular helical bundle (12). Given the structure, most antibodies raised against recombinant Glut1 protein formulated in micelles or liposomes would recognize its intracellular region. Therefore, a strategy to effectively obtain antibodies that recognize the extracellular region of the protein is needed.

Adhiron is a synthetic protein designed based on a cystatin consensus sequence, and it possesses remarkable thermal stability (melting temperature of 101 °C) (13). In previous studies, Adhiron was used as a non–immunoglobulin-binding protein, as it has two variable loops that can be replaced by random amino acid sequences to generate large phage display libraries (13, 14, 15). Importantly, one of these studies showed that a grafted sequence formed a helical structure in the designed protein, although the structure was observed in the context of a protein–protein complex (15). This result suggested to us that Adhiron could be used as a scaffold to display peptide fragments derived from large protein antigens in a manner that maintains the desired local secondary structures.

In this study, we used biophysical and computational approaches to design new antigen format proteins by grafting an extracellular helix of Glut1 onto Adhiron to maintain a local structure resembling that of Glut1. We used the designed fusion protein to generate anti-Glut1 antibodies to the extracellular region of a Glut1 membrane protein and tested whether they recognized full-length Glut1 on the cell surface. Furthermore, we also generated a VHH against glucose transporter 4 (Glut4) using the same strategy. Based on our results, we propose the possibility of designing fusion proteins as antigen formats based on characterization of the recognition mechanism of the obtained antibodies.

## Results

### Design of the scaffold to display the Glut1 peptide (amino acids 35–54)

**Figure 1:**
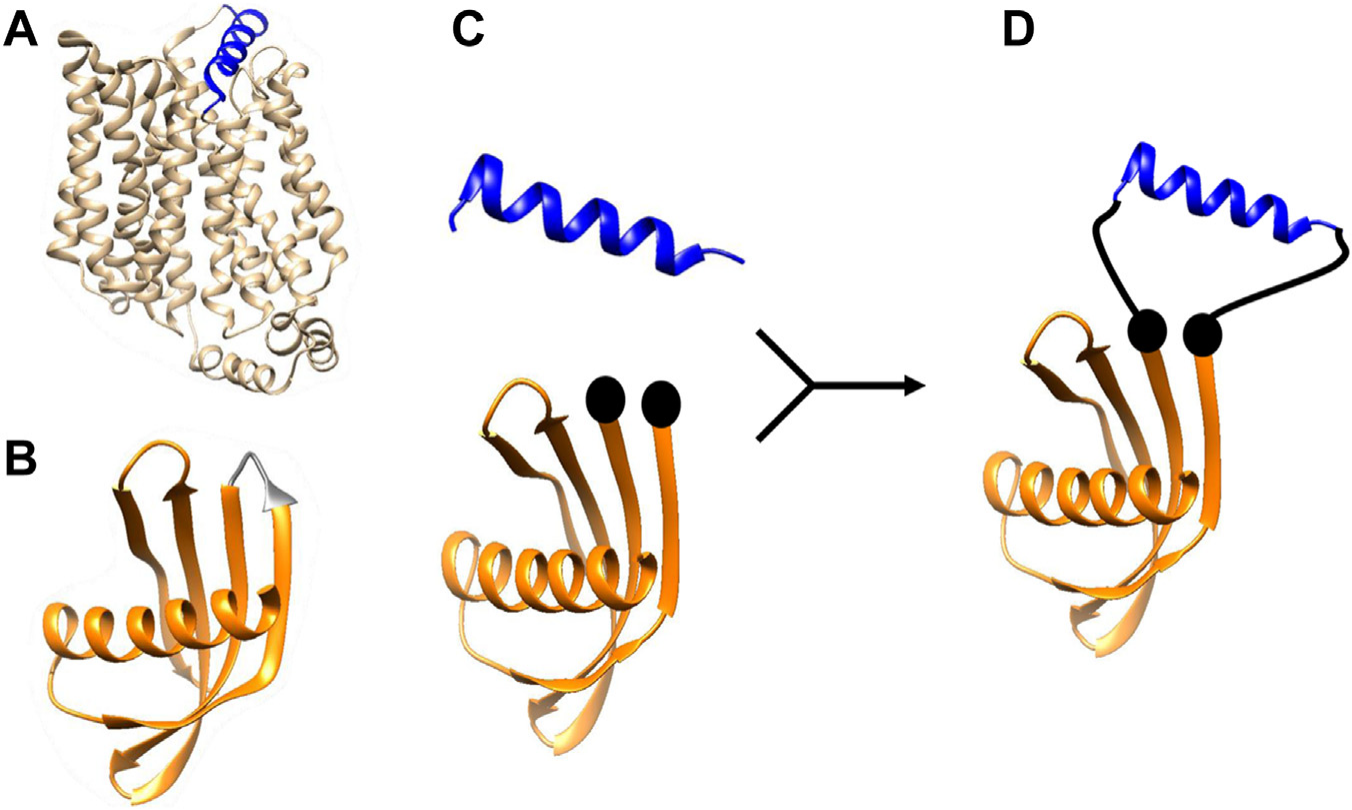
**Scheme of the protein design.***A*, Glut1 structure (Protein Data Bank ID: 4PYP). The region in *blue* is amino acids 35 to 54 of Glut1. *B*, Adhiron structure (Protein Data Bank ID: 4N6T). The region in *gray* is the loop region (_47_VVAG_50_). *C*, the structure of amino acids 35 to 54 of Glut1 (*top*). The Adhiron scaffold structure with the loop region (_47_VVAG_50_) removed (*bottom*). The two *spheres* indicate the start and the end of the loop where the grafted sequence was inserted. *D*, schematic diagram of the fusion protein. The *black lines* indicate linkers. Glut1, glucose transporter 1.

**Figure 2:**
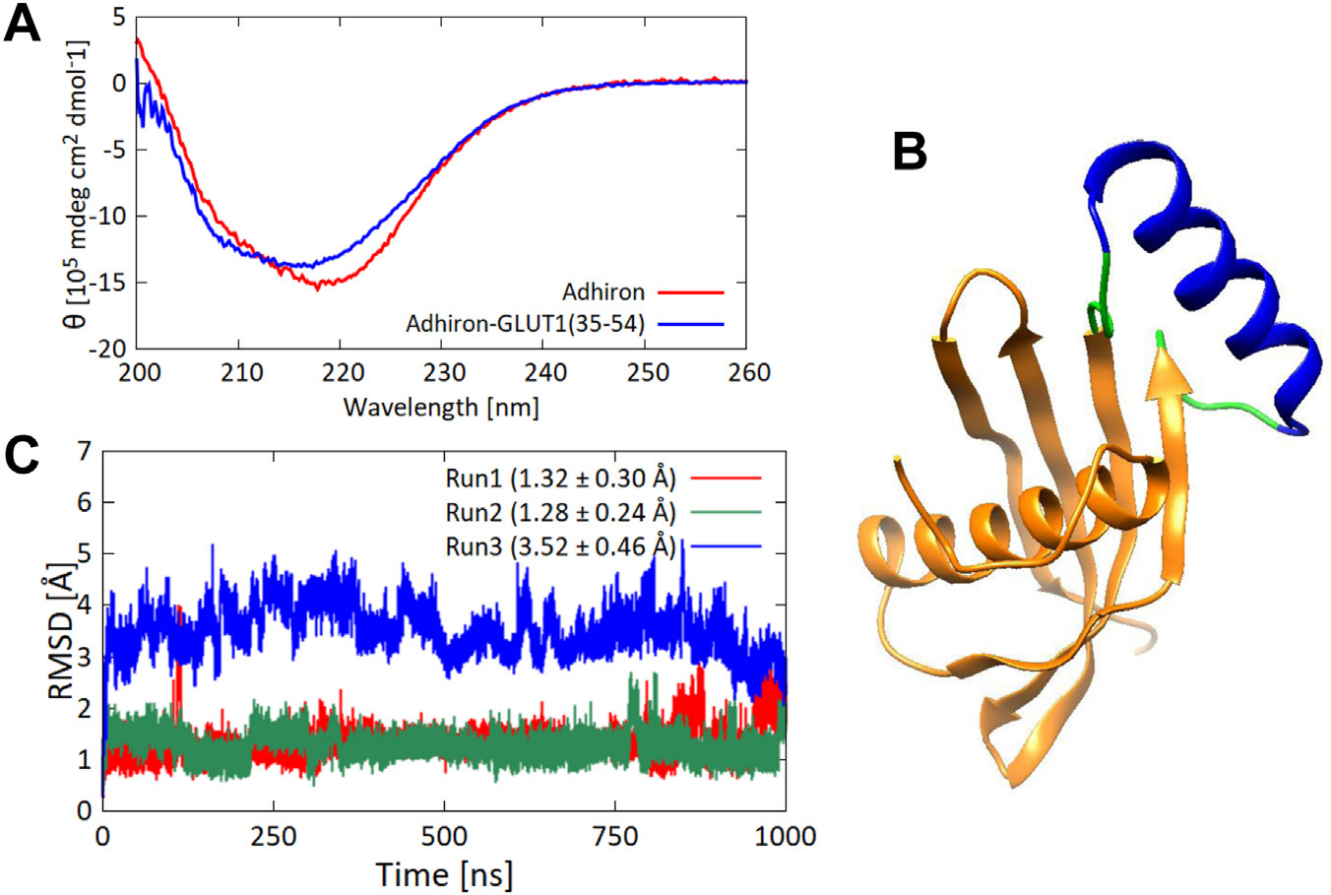
**Structural analysis of Adhiron-Glut1(35–54).***A*, CD spectra of Adhiron-WT (*red*) and Adhiron-Glut1(35–54) (*blue*). Each spectrum is the average of five measurements. *B*, model structure of Adhiron-Glut1(35–54). *C*, RMSDs of Cα atoms of Glut1 amino acids 37 to 52 between simulations and the initial structure. Each 1 μs run was performed three times, indicated in *red*, *green*, and *blue lines*. Averages and standard deviations of the RMSDs are given in *parentheses*. Glut1, glucose transporter 1.

To design a fusion protein containing the extracellular region of Glut1 in the native conformation, we grafted amino acid sequences corresponding to an extracellular α-helix of Glut1 (amino acids 35–54) into a loop of Adhiron (Fig. 1). A difference in the length of the grafted helix and the distance between terminal residues at the edge of the Adhiron loop suggested that a linker would be required to maintain the helical structure in the fusion protein (Fig. S1). We used a G4S linker (GGGGS) on either side of the grafted region. To assess whether the grafted region formed a helical structure in the fusion protein, we expressed and purified the fusion protein as well as the original Adhiron as recombinant proteins using an *E. coli* expression system and measured the CD spectra of both proteins (Fig. 2*A*). There were no increases of CD signals at 209 nm or 222 nm, which are spectral regions known to reflect the amount of α-helix structure in a protein (16). These results suggested that the grafted peptide did not assume a helical conformation in the fusion protein.

To gain molecular insights into the conformation of the grafted region, we performed molecular dynamics (MD) simulations. To prepare an initial structure for the simulation, we built a putative model structure of the fusion protein using MODELLER with the crystal structure of Adhiron (Protein Data Bank ID: 4N6T) as a template (Fig. 2*B*) (17). Rather than folding the grafted region from a linear conformation, we used the helical conformation of amino acids 35 to 54 derived from the crystal structure of full-length Glut1 (Protein Data Bank ID: 4PYP) as initial coordinates for the grafted region. We carried out triplicate 1 μs MD simulations of the fusion protein with different initial velocities and calculated RMSDs of Cα atoms of the grafted fragment, excluding two amino acids from both N and C termini (Fig. 2*C*). In one of the three trajectories, the RMSD value became larger during the simulation, implying that the grafted fragment was unstable. We then employed the DSSP program (18) to analyze the secondary structure of the grafted segment (again excluding the N- and C-terminal residues) during the simulations. We observed an α-helical conformation in 70.0% (±14.1%) of the residues during the simulations.

### Structural analysis of the grafted region in the full-length Glut1

**Figure 3:**
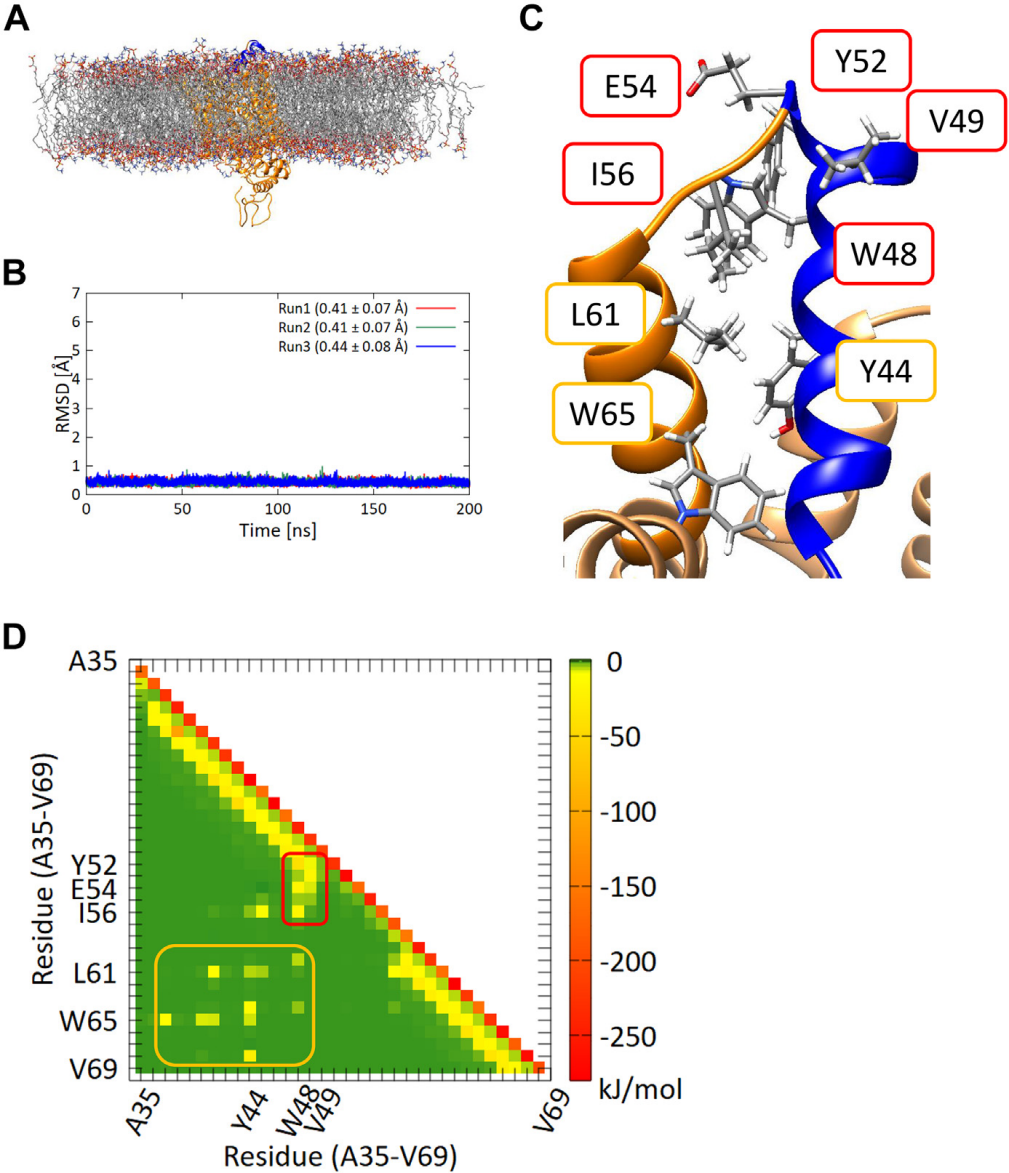
**MD simulations of the full-length Glut1.***A*, schematic of full-length Glut1 embedded in a phospholipid membrane. The region incorporated in the Adhiron fusion protein is shown in *blue*. *B*, RMSDs of Cα atoms of the 16 amino acids in the grafted region of Glut1(35–54) (excluding two residues from C and N termini) between simulations and the initial structure. Each 200 ns run was performed three times, indicated in *red*, *green*, and *blue lines*. Averages and standard deviations of the RMSDs are given in parentheses. *C*, schematic of the hydrophobic core of Glut1 showing the interaction between the grafted region (*blue*) and downstream residues. *D*, pairwise energy analysis of Glut1(35–69). Interaction energies derived from adjacent residues (*i.e.*, residues connected by a covalent bond) appear diagonally and are shown in *red*. Interaction energies derived from backbone hydrogen bonds leading to helical conformations are also on the diagonal and are shown in *yellow*. The observed hydrophobic core is boxed by a *red line*. The hydrophobic core and the interaction between the two helices are indicated by *red* and *yellow boxes*, respectively. Glut1, glucose transporter 1; MD, molecular dynamics.

The CD analysis indicated that grafting the helical region of Glut1 alone into the Adhiron loop did not induce formation of the desired helical conformation. We hypothesized that the helical conformation requires stabilization by interaction with residues outside the helical region. To visualize those interactions, we compared dynamics of the grafted region in the fusion protein with that in context of the original parent protein Glut1 by performing 200 ns MD simulations of full-length Glut1 embedded in phospholipid membranes (Fig. 3*A*). We first calculated RMSDs of Cα atoms of the helical segment (excluding two amino acids from both N and C termini) of the full-length Glut1 (Fig. 3*B*). The RMSDs were stable throughout all three trajectories and were smaller than the RMSD values for these same residues of the fusion protein Adhiron-Glut1(35–54) (Figs. 2*C* and 3*B*, respectively). In addition, assessment of the secondary structure by DSSP showed that 87.3% (±0.02%) of the peptide segment of interest adopted an α-helical conformation during the simulations, suggesting that the helical conformation is more stable in the context of the full-length Glut1 than in the context of the fusion protein. Furthermore, we observed that residues in the grafted region made hydrophobic contacts with residues close in three-dimensional space to the grafted regions (Fig. 3*C*), suggesting that these hydrophobic interactions stabilize the helical structure of the grafted region in the full-length Glut1.

To quantify these intramolecular interactions, we performed interaction energy calculations (van der Waals plus coulomb energy) for pairwise residues of the grafted region and its neighbors in the full-length Glut1 (Fig. 3*D*). Interaction energies derived from adjacent residues (*i.e.*, residues connected by a covalent bond) are strong, and those derived from backbone hydrogen bonds leading to helical conformations are moderately strong. The observed hydrophobic core is boxed by a *red line* in Figure 3*D*. These results suggest that surrounding residues stabilize the helical conformation of the grafted region in the full-length Glut1 and that the lack of these interactions in the fusion protein means that the grafted peptide segment does not form an α-helix.

### Structural analysis of a fusion protein with a longer Glut1 peptide (amino acids 35–69)

**Figure 4:**
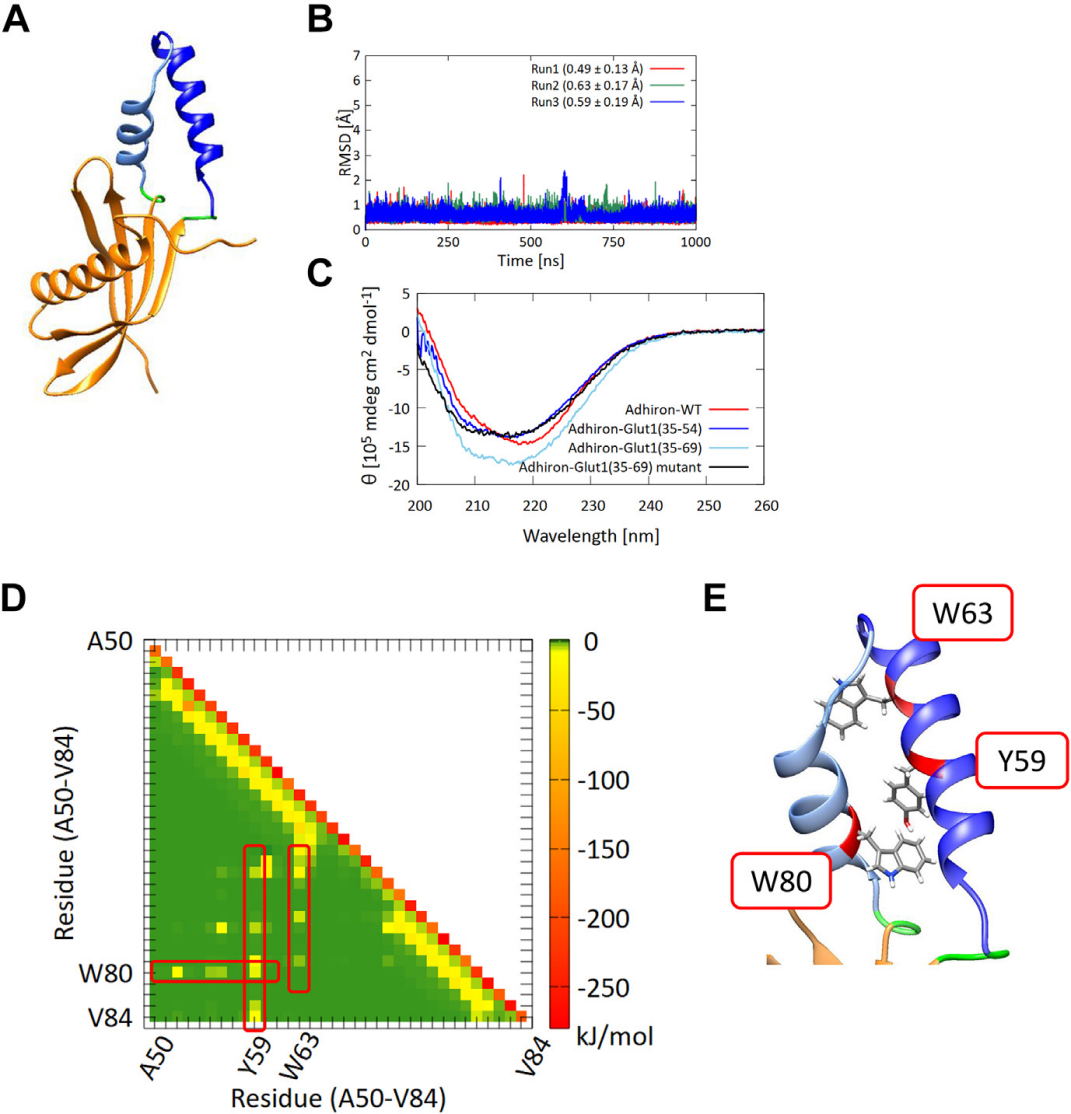
**The new Adhiron-Glut1 construct.***A*, model structure of Adhiron-Glut1(35–69). *B*, RMSDs of Cα atoms of the grafted region of Adhiron-Glut1(35–69) (excluding two residues from both C and N termini) between simulations and the initial structure. Each 1 μs run was performed three times, indicated in *red*, *green*, and *blue lines*. Averages and standard deviations of the RMSDs are given in *parentheses*. *C*, CD spectra of Adhiron-WT (*red*), Adhiron-Glut1(35–54) (*blue*), Adhiron-Glut1(35–69) (*cyan*), and Adhiron-Glut1(35–69) mutant (*black*). Each spectrum is the average of five measurements. *D*, pairwise energy analysis of the grafted region of Adhiron-Glut1(35–69). *E*, the positions of the three aromatic residues that contribute to interhelix interactions in Adhiron-Glut1(35–69). Glut1, glucose transporter 1.

Based on the results of MD simulations of full-length Glut1, we hypothesized that the interactions between residues of the helical region with those outside the helical region could stabilize the desired α-helical conformation in the fusion protein. Therefore, we designed a new Adhiron-Glut1 construct in which 35 amino acids from Glut1, including the neighboring helix region, were grafted onto Adhiron. A molecular modeling of the fusion protein suggested that the grafted region adopts an α-helical conformation (Fig. 4*A*). To verify the reliability of the model structure, we also predicted the structure of the fusion protein using ColabFold (19) (Fig. S5). The results showed that predicted structures were well accorded with the structures of the Adhiron-Glut1(35–69) modeled by MODELLER. Therefore, we used the modeled structure of the fusion protein as an initial structure in MD simulations.

The RMSDs of the Cα atoms of the Adhiron scaffold obtained from a 1 μs MD simulation indicated that the longer peptide did not alter the structure of the Adhiron scaffold (Fig. S2). This finding was confirmed by the secondary structure analysis by DSSP with visual MD (Fig. S3) (20). Compared with the grafted segment of the original fusion protein containing residues 35 to 54 of Glut1, RMSDs of Cα atoms of the grafted segment of the Adhiron-Glut1(35–69) fusion protein were smaller and more stable (Fig. 4*B*). Secondary structure analysis by DSSP showed that 86.3% (±0.7%) of the grafted segment had an α-helical conformation during the simulations, which is a considerably larger population of α-helix than observed in the original construct.

To investigate whether the grafted region forms a helical structure in the recombinant fusion protein, we measured the CD spectra of purified Adhiron-Glut1(35–69). In contrast to the initially designed fusion protein, the signals at 209 and 222 nm in the CD spectrum of Adhiron-Glut1(35–69) were significantly higher than those in the spectrum of Adhiron-WT (Fig. 4*C*), indicating that the grafted region had an α-helical conformation. Furthermore, we estimated the ratio and the number of residues for each secondary structure type in Adhiron-WT and the fusion proteins from the CD spectra by using the single spectra analysis tool available on the BeStSel server (21) (Table S4). The results showed that although the ratio of α-helical structure in Adhiron-Glut1(35–69) was less than the Adhiron-WT, the number of residues of α-helical structure in Adhiron-Glut1(35–69) was more than the Adhiron-WT, indicating that the grafted region, at least partially, had an α-helical conformation.

To further verify the importance of interhelix interactions, we calculated the interaction energies in the trajectories of the fusion protein. The interaction energy profile of Adhiron-Glut1(35–69) was similar to that of full-length Glut1 (Figs. 3*D* and 4*D*, respectively). We observed that three aromatic residues, Tyr59, Trp63, and Trp80, contributed to the interhelix interaction (Fig. 4*E*). To experimentally characterize the effects of these residues on helix formation, we prepared an Adhiron-Glut1(35–69) mutant in which these three aromatic residues were mutated to alanine. CD measurements (Fig. 4*C*) and MD simulations (Fig. S4) indicated that the grafted region of the mutant did not adopt a helical conformation. Comparison of RMSDs of Cα atoms of 16 amino acids in the grafted region Adhiron-Glut1(35–69) and the Adhiron-Glut1(35–69) mutant supports the conclusion that interhelix interactions between these three aromatic residues with the helix-forming regions stabilize the helical conformation in the context of the Adhiron fusion.

### Characterization of anti-Glut1 antibodies generated using the fusion protein

To generate anti-Glut1 antibodies, an alpaca was immunized with the fusion protein Adhiron-Glut1(35–69). After confirmation that the protein raised a serum response by ELISA, we collected a blood sample, extracted RNA from B cells, and constructed a VHH phagemid library for phage display. VHHs were selected by two rounds of biopanning on the fusion protein. In the biopanning, we used immunotubes with the immobilized scaffold protein Adhiron to subtract scaffold-specific binders. We evaluated the binding of selected VHH clones using phage VHH-ELISA (Fig. S6). The absorbance derived from the binding of VHHs to the fusion protein was compared with that of the scaffold protein Adhiron, and the DNA sequences of hit VHH clones were analyzed. The VHH clones were divided into six clusters based on the identities of the CDR sequences, and the six clones (VHH1, VHH2, VHH3, VHH4, VHH5, and VHH6) from each cluster were used for further analysis (Table S1).

To test the binding specificity of VHHs against the grafted region derived from Glut1, we prepared each VHH construct as a purified recombinant protein and measured its binding affinity to both the fusion protein and the scaffold protein using isothermal titration calorimetry (ITC). All the VHH antibodies bound to the fusion protein with nanomolar affinity. However, the VHH antibodies also bound to the scaffold protein with comparable or even higher affinity despite the subtraction steps used during biopanning (Fig. S7). The thermodynamic parameters of the interaction of each VHH to the fusion protein were similar to those of the interaction to the scaffold protein (Table S3).

**Figure 5:**
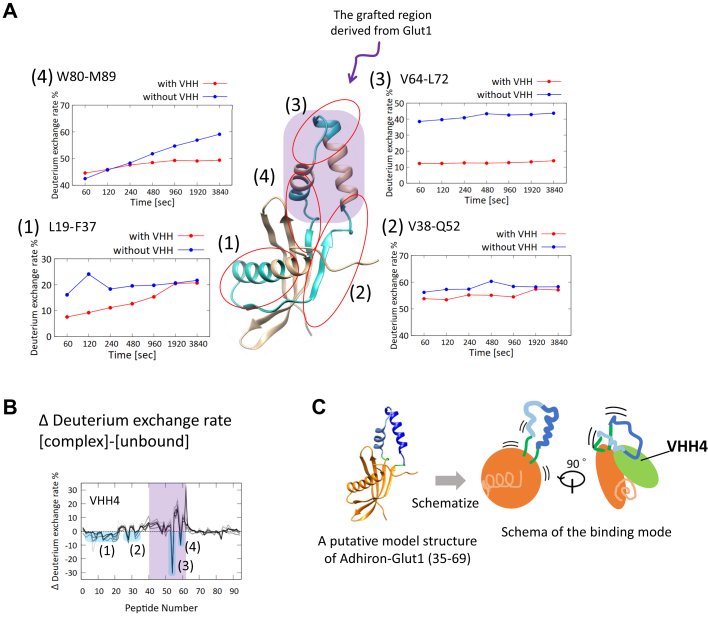
**Epitope analysis of VHH4.***A*, HDX ratio of four peptides (1) 19 to 37, (2) 38 to 52, (3) 64 to 72, and (4) 80 to 89 as a function of time. The region of these peptides is colored in *cyan* in the putative model structure of Adhiron-Glut1(35–69). The grafted region in the model structure is indicated by *purple shading*. *B*, differences of HDX ratio between the data with VHH4 and that without VHH4. The data including the four peptides of (1) 19 to 37, (2) 38 to 52, (3) 64 to 72, and (4) 80 to 89 are shaded in *cyan*. The data including the peptide in the grafted region derived from Glut1 are shaded in *purple*. *C*, schema of the binding mode of VHH4. The two grafted helices are shown in *blue* and *cyan* with *green colored* linkers. HDX, hydrogen–deuterium exchange; VHH, variable region of heavy-chain antibodies.

Although the data indicated that the VHHs recognized the scaffold region of the fusion protein, we attempted to identify the epitope region using hydrogen–deuterium exchange mass spectrometry (HDX-MS). The HDX ratios for each region of the fusion protein with or without VHH were determined, and we observed that the HDX ratio of the grafted region decreased in the presence of VHH4 and VHH5 (Figs. 5 and S8). This result suggested that the two VHHs would recognize, at least partly, the grafted region derived from Glut1 on the fusion protein (Fig. 5*C*). Therefore, these two VHH clones were chosen for further analysis.

### Recognition of full-length Glut1 by the VHHs

**Figure 6:**
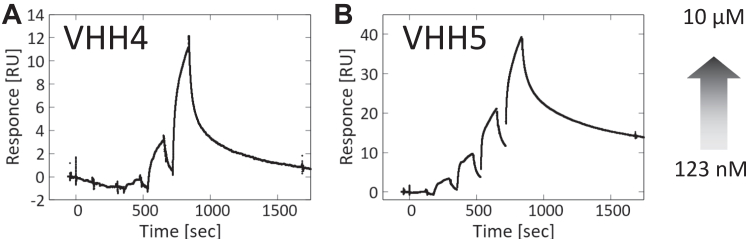
**SPR analysis of the binding of VHHs to full-length Glut1.***A*, binding of VHH4. *B*, binding of VHH5. Concentration of VHHs ranged from 123 nM to 10 μM. Glut1, glucose transporter 1; SPR, surface plasmon resonance; VHH, variable region of heavy-chain antibodies.

To determine whether the VHHs recognize full-length Glut1, we prepared full-length Glut1 as a recombinant protein and measured the binding affinity of the VHHs using surface plasmon resonance (SPR) (Fig. 6). The binding responses increased with increasing VHH concentration, indicating that both VHH4 and VHH5 bound to full-length Glut1 in micelles. However, we were unable to measure the binding parameters, presumably because the responses were too small.

**Figure 7:**
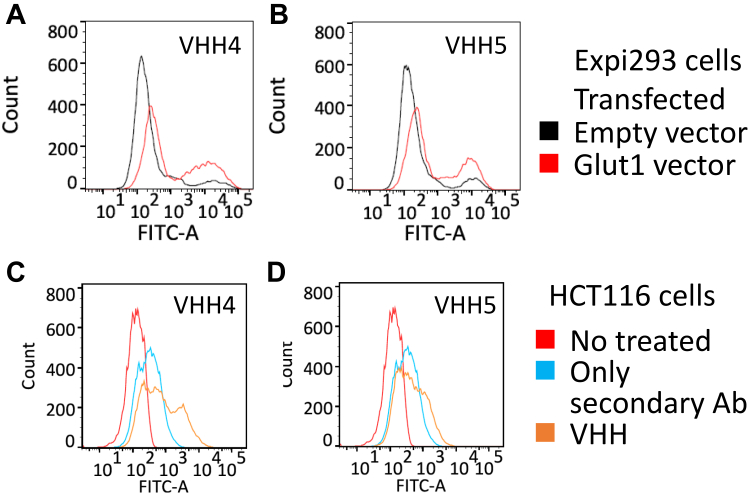
**Analysis of the binding of VHHs to Glut1 on the cell membranes.** FCM histograms for (*A* and *B*) Expi293 cells transfected with Glut1 in *red* or empty vector in *black* and (*C* and *D*) HCT116 cells. FCM, flow cytometry; Glut1, glucose transporter 1; VHH, variable region of heavy-chain antibodies.

We conducted flow cytometry (FCM) using Expi293 cells overexpressing Glut1 (Fig. S9) to validate the binding of each VHH to Glut1 on the cell membrane (Fig. 7, *A* and *B*). The fractions with high signal intensity were significantly increased by the overexpression of Glut1, indicating that the two VHHs bound to Glut1 on the cell surface. Subsequently, we used FCM using HCT116 cells derived from colon cancer to determine whether these VHHs could bind to endogenous Glut1 on the cell surface. Glut1 expression in HCT116 cells then was checked by Western blot (Fig. S10). The FCM analyses showed that the signal peaks were shifted by the addition of VHH4 or VHH5, indicating that the two clones recognized endogenous Glut1 on the surface of HCT116 cells (Fig. 7, *C* and *D*).

### Generation of anti-Glut4 VHH antibodies with the same strategy

**Figure 8:**
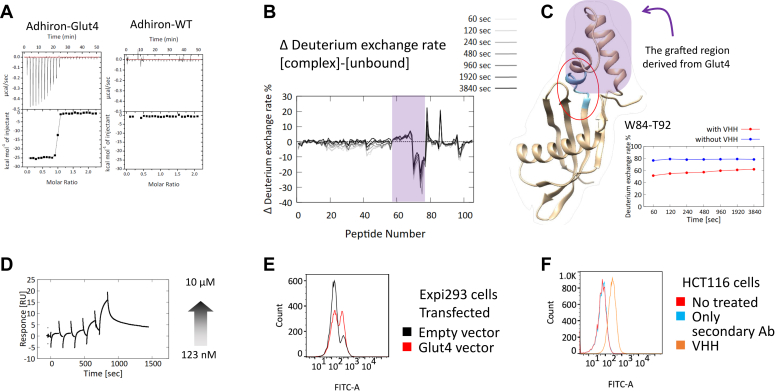
**Interaction analysis of the obtained anti-Glut4 VHH antibody.***A*, ITC results. Binding of the VHH to Adhiron-Glut4 (*left*) and to Adhiron-WT (*right*). Table S3 lists the thermodynamic parameters. *B*, differences of hydrogen–deuterium exchange ratio between the data with the VHH and that without the VHH. The data including the peptide in the grafted region derived from Glut4 are shaded in *purple*. *C*, hydrogen–deuterium exchange ratio of one peptide 84 to 92 as a function of time. The region of the peptide is colored in *cyan* in the putative model structure of Adhiron-Glut4. The grafted region in the model structure is indicated by *purple shading*. *D*, SPR analysis of the binding of the VHH to full-length Glut4. Concentration of VHH ranged from 123 nM to 10 μM. *E* and *F*, analysis of the binding of the VHH to Glut4 on cell membranes. Flow cytometry histograms for (*E*) Expi293 cells transfected with Glut4 in *red* or empty vector in *black* and (*F*) HCT116 cells. Glut4, glucose transporter 4; ITC, isothermal titration calorimetry; SPR, surface plasmon resonance; VHH, variable region of heavy-chain antibodies.

To verify how the strategy in this study is adoptable to other membrane proteins, we tried to obtain VHH antibodies against Glut4 with the same strategy. Glut4 is expressed in adipocytes, skeletal and cardiac muscles, and it functions as the insulin-responsive glucose transporter (22). In antigen design using the scaffold protein Adhiron, we chose an extracellular region containing two helices of Glut4 as a grafted region, as well as Glut1 and called Adhiron-Glut4. We immunized alpaca with Adhiron-Glut4 and selected VHH antibodies using the phage display method, resulting in acquisition of one VHH antibody, which bound to Adhiron-Glut4. Remarkably, the obtained VHH bound only to Adhiron-Glut4 and not to the scaffold protein (Fig. 8*A*). Epitope analysis using HDX-MS showed that the VHH bound to the grafted region including the linker (Fig. 8, *B* and *C*). In addition, to check the binding of the VHH to full-length Glut4 as a recombinant protein, we performed SPR measurement and confirmed the binding (Fig. 8*D*). Furthermore, the data of FCM showed that the VHH recognized both overexpressed Glut4 on the Expi293 cells and endogenous Glut4 on the HCT116 cells (Fig. 8, *E* and *F*). Altogether, we successfully obtained a VHH antibody against Glut4, guaranteeing our strategy.

## Discussion

In this study, we designed fusion proteins consisting of Adhiron and an extracellular region of Glut1, which has an intrinsic helical structure, to generate site-specific antibodies. By using the fusion protein, we successfully obtained several VHH antibodies that recognize full-length Glut1 at both molecular and cellular levels, which suggests that we succeeded in mimicking the local structure of the extracellular region of Glut1 by fusion with the scaffold protein Adhiron.

When designing the fusion protein, the combination of biophysical analysis and MD simulations revealed the importance of intramolecular interactions in maintaining the desired helical conformation of the grafted region. It should be noted that although CD spectra of the initial fusion proteins showed that the grafted region did not assume a helical conformation, the results of MD simulations showed that the grafted region was helical about 70% of the time. The discrepancy between *in vitro* and *in silico* analyses was likely because of intrinsic features of the MD simulations: Dynamics of proteins in simulations strongly depend on the starting structure (which was an α-helix in this study), and the force field used (CHARMM) tends to overestimate the formation of α-helical structure (23, 24). Our MD simulations showed that the fusion protein containing additional Glut1 residues formed α-helical conformations more often than did the initial fusion protein (approximately 87% *versus* 70% of the simulation times, respectively), which qualitatively agreed with the CD results. Simple RMSD-based prediction was useful in designing the fusion proteins in this study, and optimization of force-field parameters will enable more quantitative predictions of protein dynamics and formation of secondary structures.

We used the fusion proteins for both alpaca immunization and subsequent phage display–based selection with subtraction using the parent scaffold protein to isolate the VHH antibodies that recognizes the grafted region specifically. The ITC measurement and HDX-MS analysis results showed that all six of the selected VHH clones bound to the scaffold region despite the subtraction step taken during VHH selection, although two of these clones (VHH4 and VHH5) also recognized the grafted region. These findings highlight the difficulty of designing fusion proteins as well as the need to consider experimental settings. Taken together with the SPR results showing that use of the full-length Glut1 in micelles resulted in a quite small response, this combination of results indicated that the affinity of the VHH clones toward Glut1 was not high and that the epitope region for each VHH was largely distributed in the scaffold region, which is consistent with HDX-MS results. VHH1, which did not recognize the grafted region, shares many amino acid sequences in each CDR loop with the two hit clones (Table S1), which indicates that only a few amino acids are responsible for recognition of the grafted region. Identification of such amino acid residues will provide the basis for affinity maturation of generated VHH antibodies.

Finally, we used FCM to validate the binding of the VHHs to Glut1 on the cell surface. VHH4 and VHH5 recognized overexpressed Glut1 and endogenous Glut1 on the plasma membrane. The distribution pattern of peak fractions for Expi293 cells and HCT116 cells differed for each VHH clone. It is possible that each VHH recognizes a distinct molecular state of Glut1. Because major facilitator superfamily transporters, including those in the Glut family, must undergo conformational changes in order to transport their substrates across biological membranes (25), Glut1 is predicted to have several states on the cell membranes. Further analysis of the molecular mechanism by which each VHH recognizes the full-length Glut1 may lead to a better understanding of the structural dynamics of Glut1 on the cell surface.

In addition to anti-Glut1 antibodies, we attempted to obtain anti-Glut4 VHH antibodies with the same approach and successfully isolated a VHH antibody, which was able to recognize endogenous Glut4 on the cell membrane. It is noteworthy that the obtained VHH did not bind to the scaffold protein but only did bind to the fusion protein. It might be because the targeted region of Glut4 has different dynamics from that of Glut1.

In summary, we successfully designed a fusion protein that displays an extracellular helix from the membrane protein Glut1/Glut4 and generated antibodies that were able to recognize Glut1/Glut4 on the cell surface using the designed protein. This strategy can be applied to generate antibodies to a particular epitope with a certain conformation.

## Experimental procedures

### Expression and purification of Adhiron-WT and the designed proteins

The coding sequence of Adhiron was inserted into a modified pET28b vector with a His6 tag and a SUMO tag at the N terminus. The plasmid was transformed into BL21(DE3) *E. coli* cells and cultured at 28 °C in lysogeny broth medium containing 50 μg/ml kanamycin. When the culture reached an absorbance at 600 nm of approximately 0.6, protein synthesis was induced by adding 0.5 mM isopropyl-ß-d-1-thiogalactopyranoside, and cells were cultured at 20 °C for 18 h.

Cells were harvested by centrifugation at 7000*g* for 10 min at 4 °C and disrupted by sonication in binding buffer composed of 20 mM Tris–HCl (pH 8.0), 5 mM imidazole, and 100 mM NaCl. The lysates were centrifuged at 40,000*g* for 30 min at 4 °C, and the supernatants were loaded onto a nickel–nitrilotriacetic acid (Ni–NTA) agarose (QIAGEN) column that had been pre-equilibrated with binding buffer at 4 °C. The column was washed with five column volumes of the binding buffer. The protein of interest was eluted with five column volumes of an elution buffer composed of 20 mM Tris–HCl (pH 8.0), 500 mM NaCl, and imidazole. The concentration of imidazole was 50 mM for Adhiron-WT and 200 mM for the designed proteins.

The protein fractions were dialyzed overnight at 4 °C in binding buffer containing protease Ulp1. After dialysis, the samples were again passed through a Ni–NTA column. Finally, the flow-through samples were purified over a HiLoad 26/60 Superdex 75 pg size-exclusion column (Cytiva). The column was equilibrated with PBS at 4 °C. The elution profile was monitored at 280 nm, and the concentration of each protein was determined by measuring the absorbance at 280 nm.

### CD spectra

CD spectra were recorded on a model J-820 CD spectrometer (JASCO). Far-UV CD measurements were performed with 10 μM of protein in PBS using a 0.1 cm cell and a bandwidth of 1 nm. Spectra were recorded five times for each sample, and the results were averaged.

### MD simulations

MD simulations were performed using GROMACS 2016.3 (26) with the CHARMM36m force field (24). Using the CHARMM-GUI (27), designed proteins and Adhiron-WT were solvated with TIP3P water in a rectangular box such that the minimum distance to the edge of the box was 15 Å under periodic boundary conditions. Full-length Glut1 was placed in the center of a 1-palmitoyl-2-oleylphosphatidylcholine lipid bilayer and subsequently solvated with TIP3P water in a 150 × 150 × 150 Å box. Sodium and chloride ions were added to neutralize the protein charge, and then more ions were added to mimic a salt solution concentration of 0.14 M. Each system was energy-minimized for 5000 steps and equilibrated with NVT ensemble at 298 K for 1 ns. Further production runs were performed for 1000 and 200 ns with NPT ensemble for designed proteins and full-length Glut1, respectively. A cutoff distance of 12 Å for Coulomb and van der Waals interactions was used.

Long-range electrostatic energies were evaluated using the particle mesh Ewald method (28). The LINCS algorithm was employed to constrain bonds involving hydrogen atoms (29). The time step was set to 2 fs throughout the simulations. Simulations were repeated three times for each system with different velocities, and snapshots were saved every 10 ps. UCSF Chimera (30) was employed to analyze and visualize the MD trajectories and to render the molecular graphics.

### Alpaca immunization followed by library construction and selection

An alpaca was immunized with the designed fusion protein. Library construction from the peripheral blood B cells obtained from the immunized alpaca was conducted as described in a previous study (31). Briefly, total RNA was obtained using Trizol and used for complementary DNA synthesis. Antibody genes were amplified by PCR and incorporated into a phagemid vector. The library DNA was electroporated into *E. coli* XL-1 Blue followed by VCS M13 helper phage infection, and phage production was induced in the presence of 1 mM of isopropyl-1-thio-β-d-galactopyronoside. Phages were precipitated from the bacterial supernatant with polyethylene glycol/NaCl and resuspended in PBS. The VHH antibodies were selected by two rounds of biopanning using immunotubes, and selected antibody sequences were analyzed.

### VHH preparation as recombinant proteins

Gene fragments encoding VHH with His tag at the C terminus were cloned into pRA2 vector for bacterial expression. Expression and purification of the recombinant VHHs were carried out based on a previous report (32). VHHs were expressed using BL21(DE3) *E. coli*. Isopropyl-1-thio-β-d-galactopyronoside (0.5 mM) was added to the growing cells when an absorbance at 600 nm value reached ∼1.0 to induce protein expression. Cells were harvested after overnight culture.

The *E. coli* pellet was resuspended in 20 mM Tris–HCl (pH 8.0), 500 mM NaCl, and 5 mM imidazole, and then the cells were disrupted by sonication. Immobilized metal affinity chromatography was conducted using Ni–NTA agarose. The proteins were eluted with 20 mM Tris–HCl (pH 8.0), 500 mM NaCl, and 200 mM imidazole. The proteins were dialyzed against a PBS solution (pH 7.4). The final purification was performed by size-exclusion chromatography using a HiLoad 26/600 Superdex 75 pg column (Cytiva) at 4 °C equilibrated in PBS (pH 7.4). The monomer peak fractions were collected, and the purity of each VHH was evaluated by SDS-PAGE followed by Coomassie staining.

For SPR analysis, we also prepared recombinant VHHs with a FLAG tag. Gene fragments encoding VHH with FLAG tag at the C terminus were cloned into pRA2 vector for bacterial expression. The procedure for expression and purification was the same as that used for VHH with His tag until the sonication step. The cell lysate was centrifuged at 40,000*g* for 30 min at 4 °C. The supernatant was filtered through an 0.8 μm pore-size filter and subsequently loaded onto a DDDDK-tagged Protein Purification Gel (MBL) equilibrated with 20 mM Tris–HCl (pH 8.0), 500 mM NaCl, and 5 mM imidazole. The proteins were eluted with 1 M arginine (pH 4.4). The eluate was immediately neutralized with 1 M Tris–HCl (pH 8.0). The proteins were dialyzed against a PBS solution (pH 7.4). The final purification was performed by size-exclusion chromatography.

For FCM analysis, the gene encoding the obtained VHH antibodies was subcloned into pcDNA 3.4, which is an expression vector for mammalian expression systems (Thermo Fisher Scientific) that contains an Igκ signal peptide sequence and Fc region of trastuzumab at the C terminus. ExpiCHO cells (Thermo Fisher Scientific) were transfected with the expression vectors of Fc-VHH antibodies, and the supernatant was collected 14 days after transfection. The supernatant was loaded on rProtein A Sepharose Fast Flow (Cytiva) equilibrated with PBS. The resin was washed with PBS, and subsequently, the Fc-VHH antibodies were eluted using IgG Elution Buffer (Thermo Fisher Scientific). The eluted fractions were immediately neutralized with 1 M Tris–HCl (pH 8.0) and further purified by size-exclusion chromatography using a HiLoad 26/600 Superdex 200-pg column equilibrated with PBS.

### ITC

The binding of the obtained VHH antibodies to the fusion protein was evaluated by ITC. To the solution of 10 μM VHH antibody in the cell, 2 μl of the solution containing 100 μM of the fusion protein were titrated by injection 20 times at the interval of 120 s with the stirring rate at 750 rpm. The measurements were conducted in PBS (pH 7.4) at 25 °C.

### HDX-MS

We used HDX-MS to identify the epitope region of the obtained VHH antibodies. We prepared the fusion protein and VHH antibodies at 70 μM in H_2_O-based PBS. The HDX reaction was started by diluting D_2_O-based buffer 10-fold at 10 °C, and it was quenched by adding an equal volume of prechilled quenching buffer (8 M urea, 1 M Tris(2-carboxyethyl)phosphine hydrochloride, pH 3.0) using HDx-3 PAL (LEAP Technologies). The quenched protein samples were subjected to online pepsin digestion and analyzed by liquid chromatography–MS using an UltiMate3000RSLCnano device (Thermo Fisher Scientific) connected to the Q Exactive plus mass spectrometer (Thermo Fisher Scientific). Online pepsin digestion was performed using a Poroszyme Immobilized Pepsin Cartridge (2.1 × 30 mm; Waters) and a protease type XIII/pepsin column (w/w, 1:1; 2.1 × 30 mm; NovaBioAssays) in formic acid solution (pH 2.5) at 8 °C for 3 min at a flow rate of 50 μl/min. The desalting column and the analytical columns were Acclaim PepMap300 C18 (1.0 × 15 mm; Thermo Fisher Scientific) and Hypersil Gold (1.0 × 50 mm; Thermo Fisher Scientific), respectively. The mobile phases were 0.1% formic acid solution (A buffer) and 0.1% formic acid containing 90% acetonitrile (B buffer). The deuterated peptides were eluted at a flow rate of 45 μl/min with a gradient of 10 to 90% of B buffer in 9 min.

MS conditions were as follows: an electrospray voltage of 3.8 kV, positive ion mode, sheath and auxiliary nitrogen flow rate at 20 and two arbitrary units, ion transfer tube temperature at 275 °C, auxiliary gas heater temperature at 100 °C, and a mass range of *m/z* 200 to 2000. The data-dependent acquisition was performed with normalized collision energy of 27 arbitrary units. The MS and tandem MS spectra were subjected to a database search analysis using Proteome Discoverer 2.2 (Thermo Fisher Scientific) against an in-house database containing the amino acid sequence of the fusion protein. We used HDExaminer software (Sierra Analytics) and the MS raw files to analyze the deuteration levels of the peptide fragments by comparing the spectra of deuterated samples with those of nondeuterated samples. Data summary for each HDX-MS was shown in Table S2.

### Preparation of Glut1/Glut4 as a recombinant protein

The gene encoding human Glut1/Glut4 was subcloned into a pFastBac1 vector (Invitrogen) with a C-terminal 10× His tag. Bacmids were prepared according to the manufacturer’s protocol (Bac-to-Bac Baculovirus Expression System; Thermo Fisher Scientific). Sf9 insect cells (Thermo Fisher Scientific) were transfected with Glut1/Glut4 bacmids, and the produced baculo virus was amplified according to the manufacturer’s protocol. For expression of Glut1 and Glut4, 1.8 × 10^6^ cells/ml of High Five Insect Cells (Thermo Fisher Scientific) were infected with the virus and incubated with shaking at 120 rpm at 27 °C for 72 h.

The purification of recombinant Glut1 and Glut4 was conducted following the method described in a previous study (12). Briefly, the cells were harvested by centrifugation and disrupted using a Dounce homogenizer on ice. Cell debris was removed by centrifugation (1000*g* for 10 min at 4 °C) followed by ultracentrifugation. Membrane fractions were homogenized and solubilized with a buffer containing 2% (w/v) *n*-dodecyl-β-d-maltopyranoside. After ultracentrifugation, the supernatant was purified using Ni–NTA agarose followed by size-exclusion chromatography.

### SPR using recombinant Glut1/Glut4

The binding of the obtained VHHs to full-length Glut1/Glut4 as a recombinant protein was analyzed using a Biacore 8K instrument (Cytiva). Anti-His antibodies, bovine serum albumin–free antibodies (QIAGEN), were immobilized on a CM5 Biacore sensor chip (Cytiva) following the manufacturer’s protocol. Glut1/Glut4 was captured by anti-His antibodies. After confirming the equilibration, VHHs were injected, and a range of concentrations (123 nM–10 μM) was tested. The association time was 120 s, and the dissociation time was 2500 s. The assays were carried out in 25 mM Mes–NaOH (pH 6.0), 150 mM NaCl, 5% (w/v) glycerol, 0.05% (w/v) n-dodecyl-β-d-maltoside at 25 °C. The data were collected in single-cycle kinetics mode.

### Western blotting

Expi293 and HCT116 cells were purchased from ThermoFisher Scientific and JCRB Cell bank, respectively. Cell lysate samples from Expi293 overexpressing Glut1 or HCT116 were prepared with radioimmunoprecipitation assay cell lysis buffer (Santa Cruz) supplemented with cOmplete Protease Inhibitor Cocktail (Roche Applied Science). Total amount of proteins was determined using BCA Protein Assay Kit (ThermoFisher Scientific), and whole-cell lysates were separated by SDS-PAGE and blotted to nitrocellulose membrane. Protein bands were detected by anti β-actin or anti-Glut1 antibody followed by the incubation with horseradish peroxidase–conjugated anti-rabbit IgG, horseradish peroxidase–linked antibody (Cell Signaling Technology) at room temperature for 30 min, and visualizing with enhanced chemiluminescence (GE Healthcare). The membrane image was captured using LAS500 (Cytiva).

### FCM

To validate the binding of the obtained VHH antibodies to Glut1 and Glut4 on the cell surface, we conducted FCM using a BD FACSCanto II (BD Biosciences). For cell preparation, the gene encoding human Glut1/Glut4 was cloned into pcDNA 3.4. Expi293 cells were transfected with the vector and harvested 3 days after the transfection. As a negative control, we prepared Expi293 cells transfected with an empty vector. We used Goat Antihuman IgG (H+L) Cross-Adsorbed Secondary Antibody and Alexa Fluor 488 (Thermo Fisher Scientific). The cells were washed twice with FACS buffer (PBS, 2% [v/v] fetal bovine serum) and then coincubated simultaneously with 1 μM Fc-VHH antibody and the secondary antibody on ice for 60 min. The cells then were washed twice with FACS buffer and analyzed. Collected data were analyzed using FlowJo software (BD Biosciences).

## Data availability

All the data obtained in this research are included in the article or supporting information.

## Supporting information

This article contains supporting information.

## Conflict of interest

The authors declare that they have no conflicts of interest with the contents of this article.
