# Supplementary material for: Generation of antibodies to an extracellular region of the transporters Glut1/Glut4 by immunization with a designed antigen
Source: J Biol Chem. 2024 Jan 9;300(2):105640. doi: 10.1016/j.jbc.2024.105640 (PMC10862009; doi:10.1016/j.jbc.2024.105640)
Supplement: Supplementary file 1 [file mmc1.pdf]

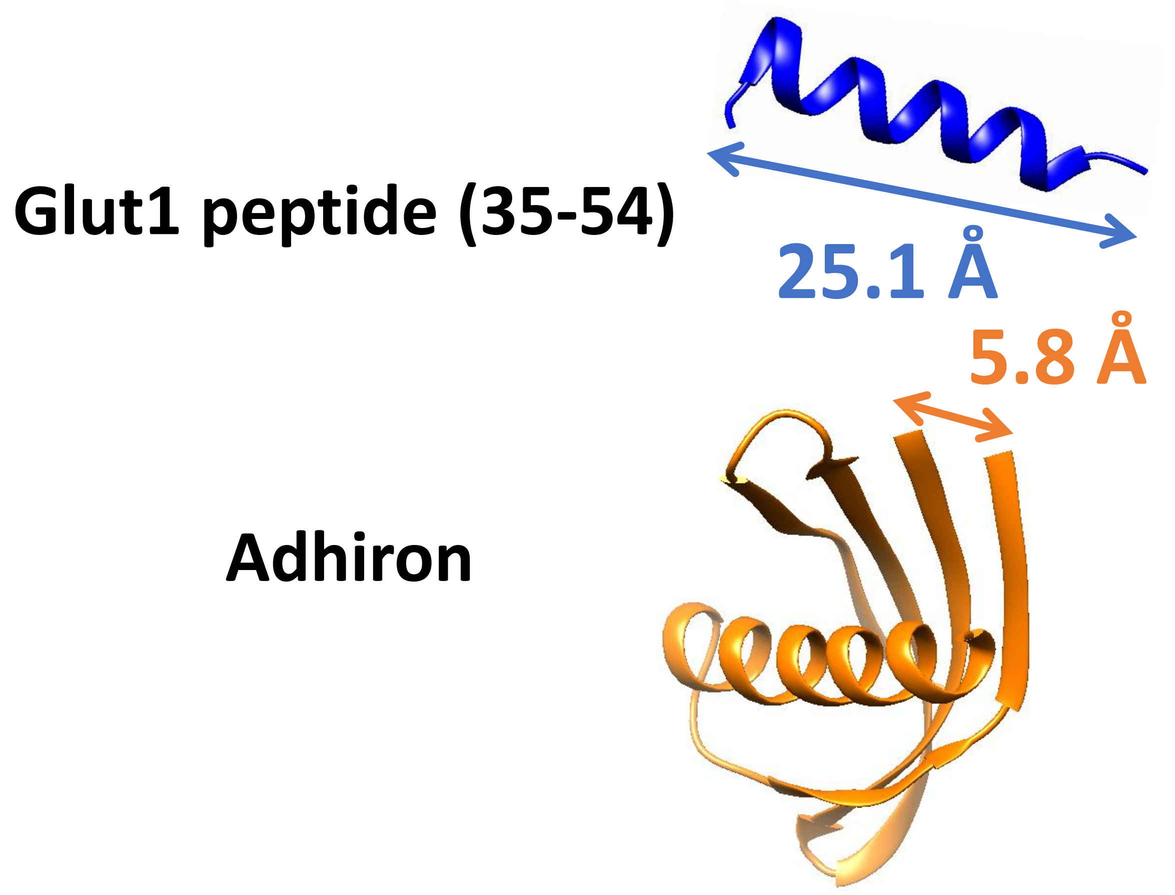

Figure S1.
Length of the grafting helix, and the distance between amino acid residues on either edge of the Adhiron loop.

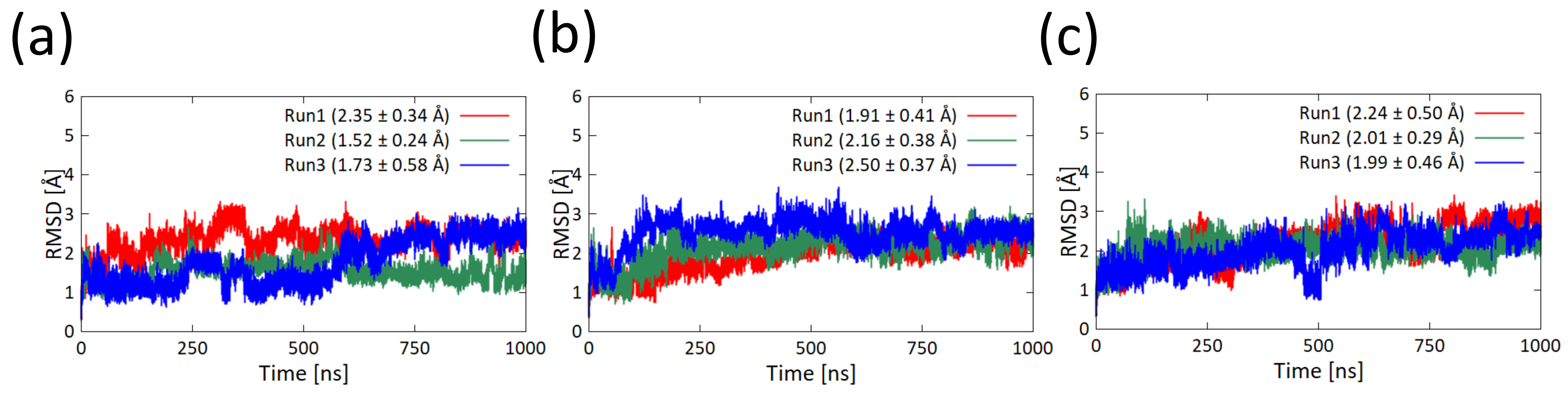

Figure S2

RMSDs of Cα atoms of (A) Adhiron-Glut1(35–54), (B) Adhiron-Glut1(35–69), and (C) wild-type Adhiron between simulations and the initial structure. Each 1-μs run was performed three times, indicated in red, green, and blue lines. Averages and standard deviations of the RMSDs are given in parentheses.

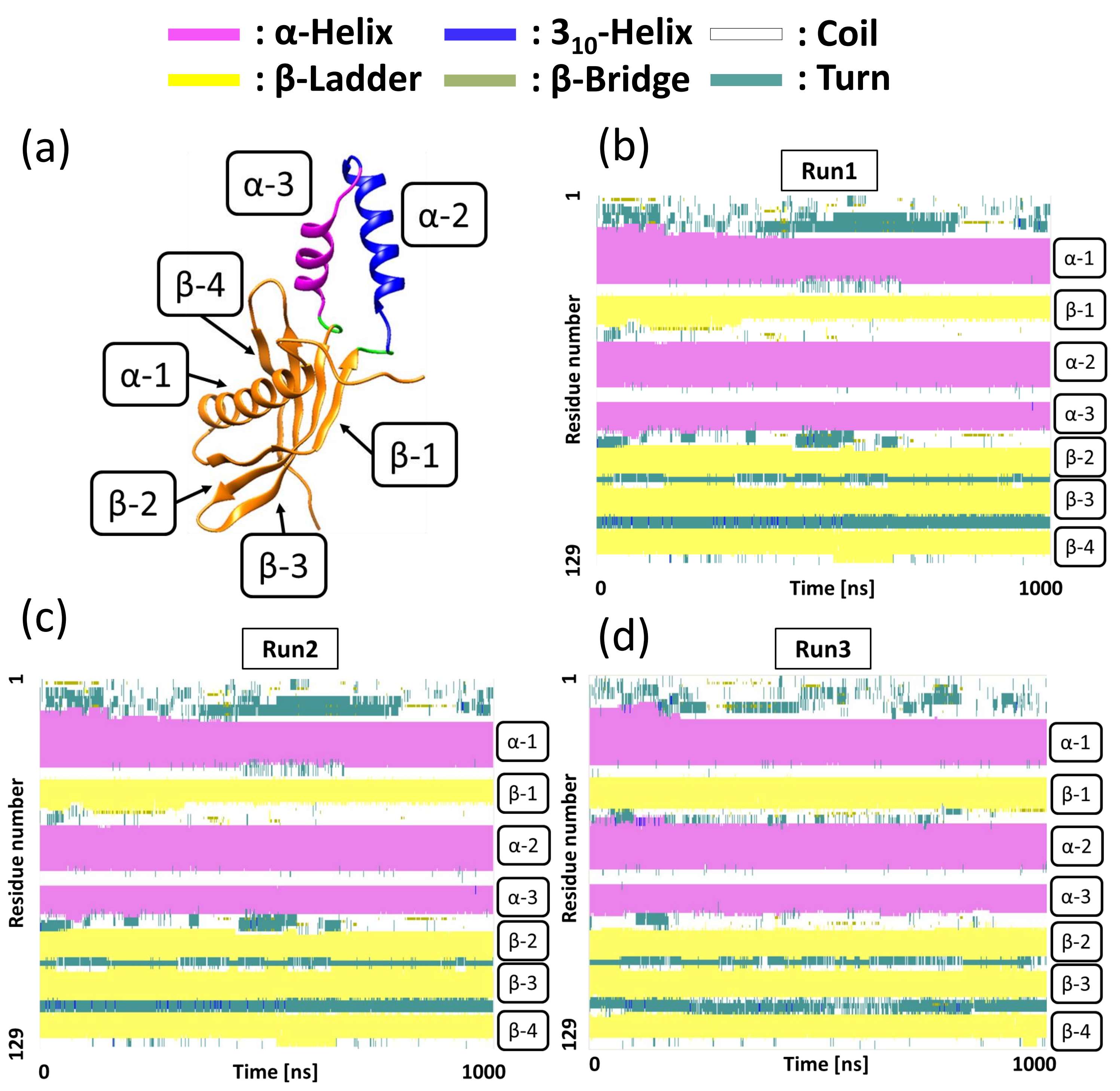

Figure S3

(A) secondary structures (i.e., three α-helices and four β-strands) of the model structure of Adhiron-Glut1(35–69). (B–D) time course of the secondary structure of whole Adhiron-Glut1(35–69) in each run as determined by DSSP.

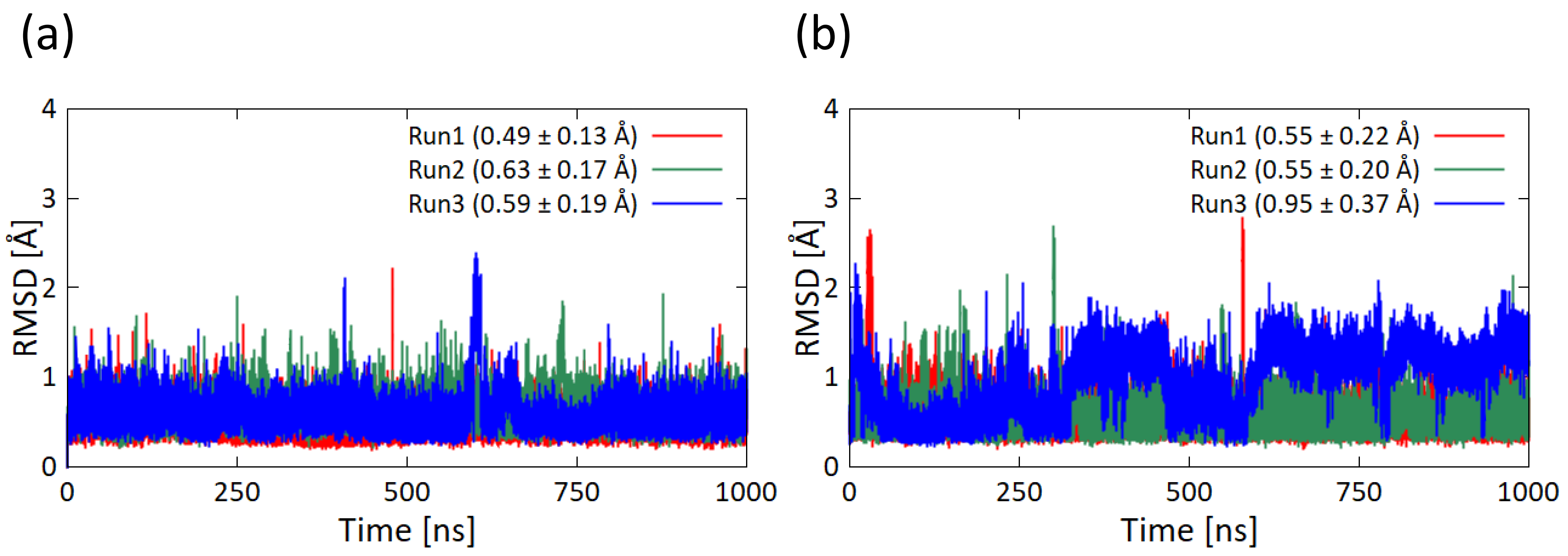

Figure S4

Comparison of RMSDs of Cα atoms of 16 amino acids of the grafted region of Adhiron-Glut1(35–69) and the Adhiron-Glut1(35–69) mutant.

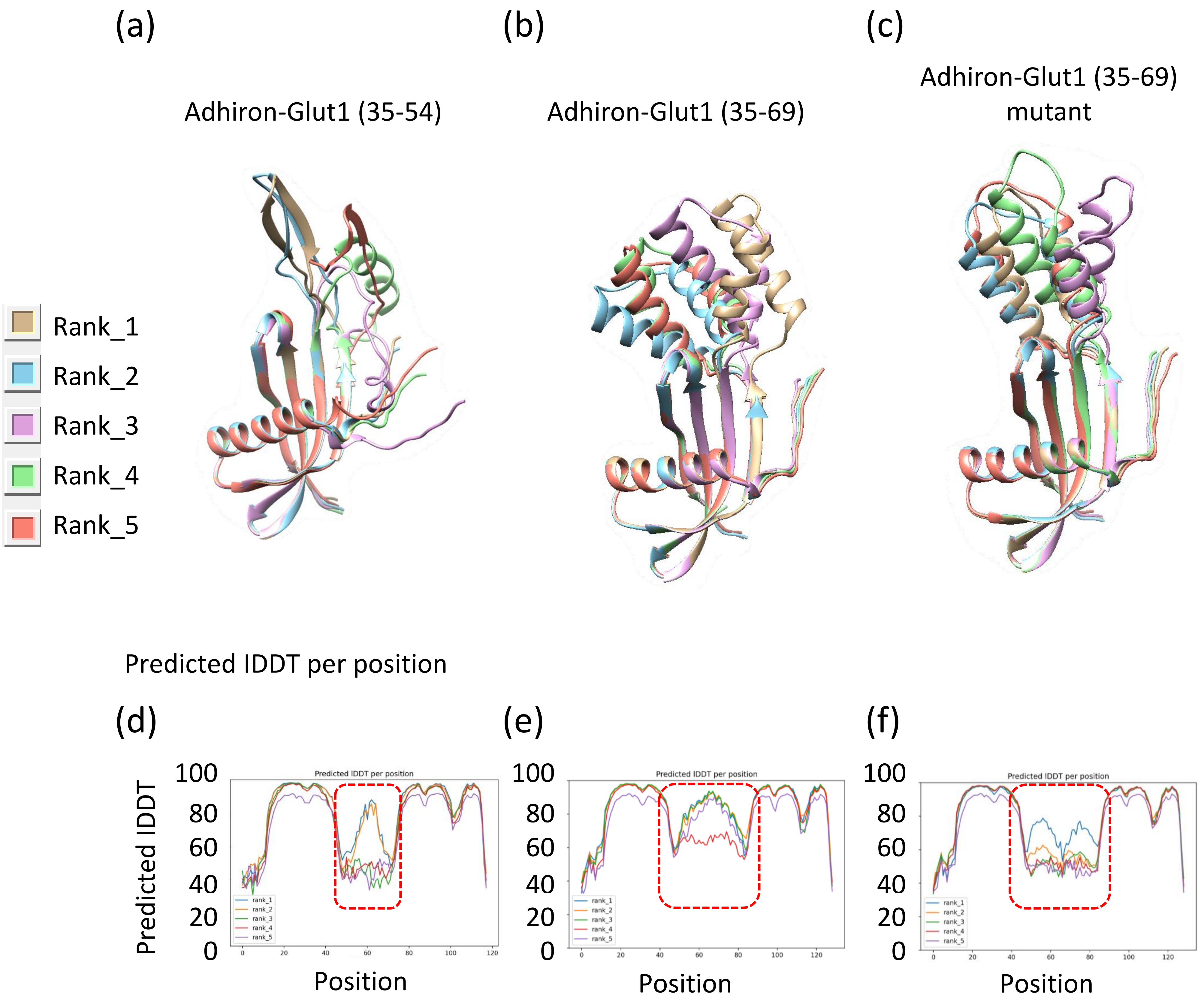

Figure S5

(A–C) predicted structures using Colabfold. (A) Adhiron-Glut1(35–54), (B) Adhiron-Glut1(35–69), and (C) Adhiron-Glut1(35–69) mutant. (D–F) predicted IDDT per position. The grafted region was boxed by a red dotted line. (D) Adhiron-Glut1(35–54), (E) Adhiron-Glut1(35–69), and (F) Adhiron-Glut1(35–69) mutant.

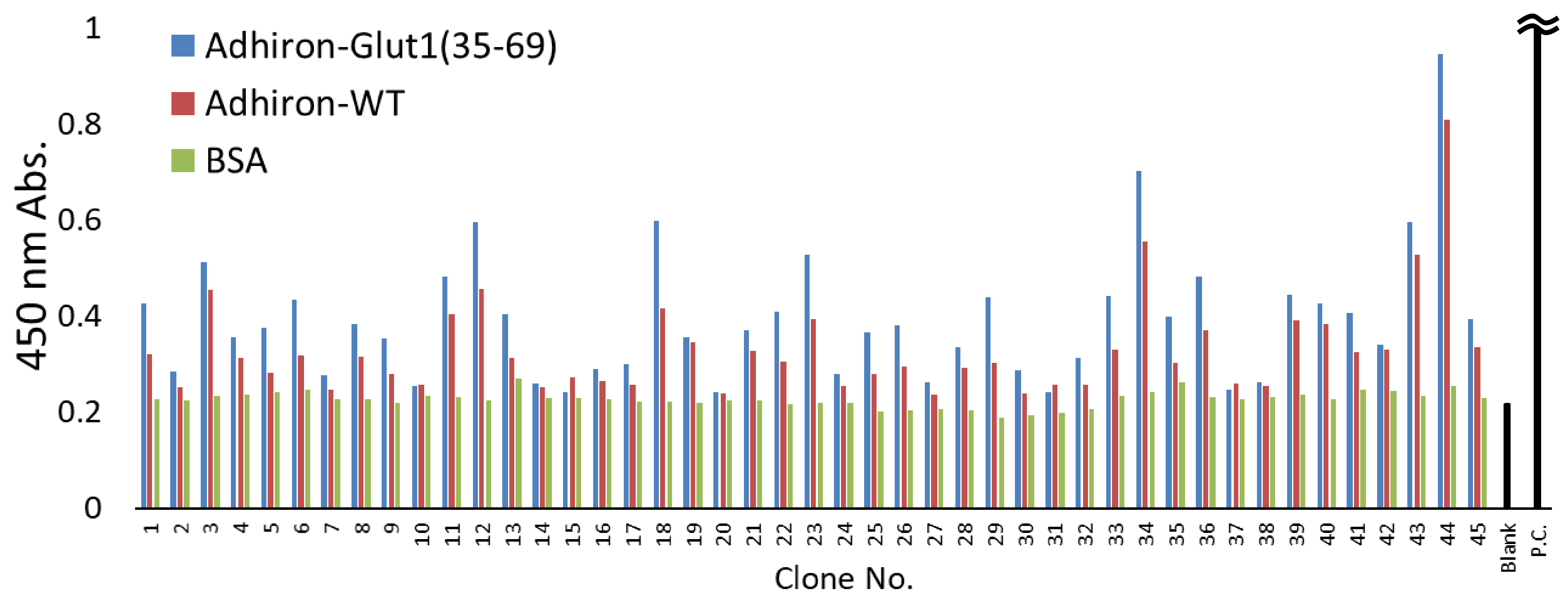

Figure S6

Result of phage ELISA. The blue bar corresponds to the absorbance derived from the binding of the fusion protein. The red bar corresponds to the absorbance derived from the binding of the scaffold protein Adhiron. The green bar corresponds to the absorbance derived from the binding of BSA, as a control experiment. Table S1 lists the sequences of the selected antibodies for the subsequent analyses.

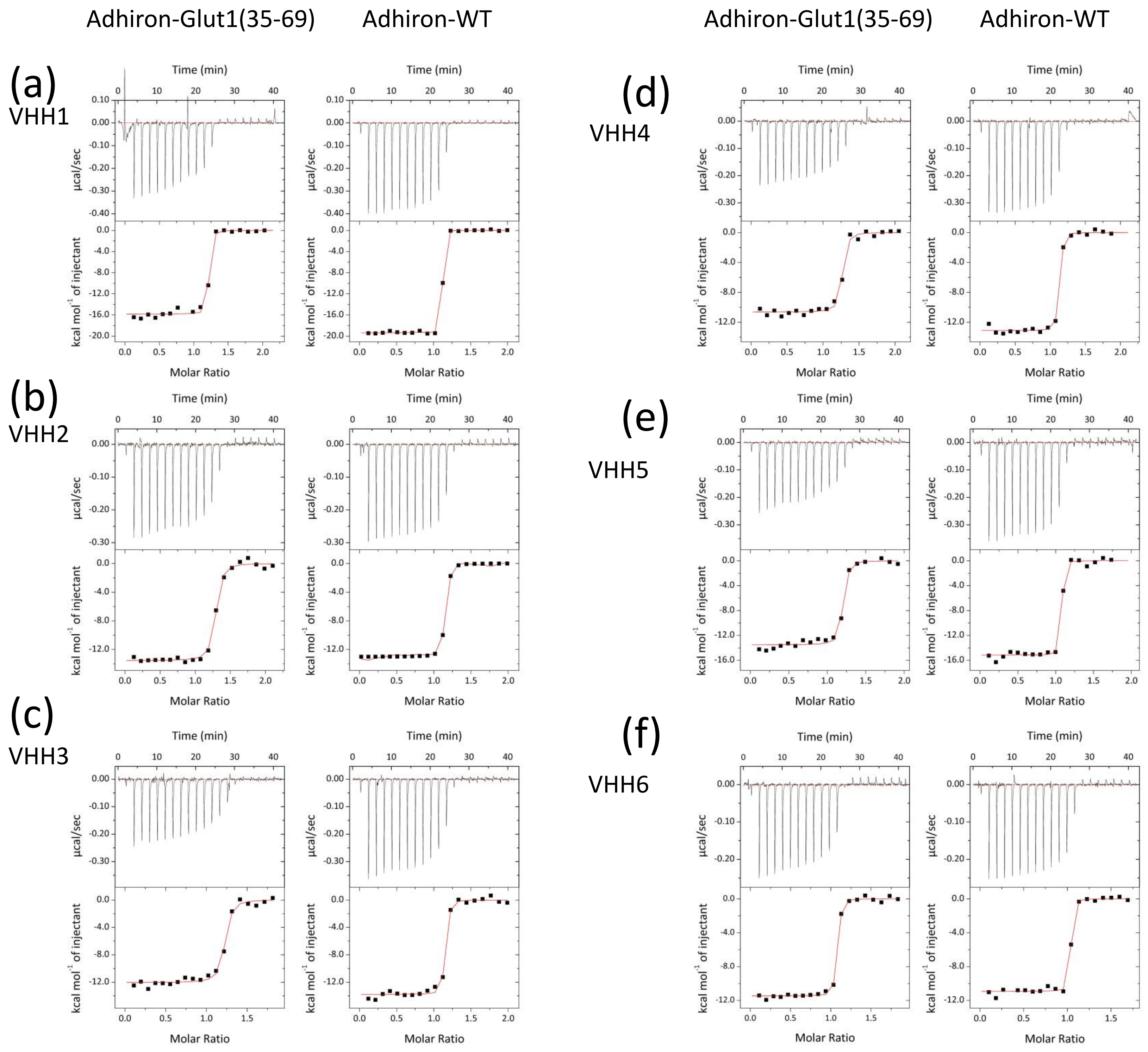

Figure S7

ITC results. Binding of VHHs to Adhiron-Glut1(35–69) (left) and to Adhiron-WT (right). Table S3 lists the thermodynamic parameters.

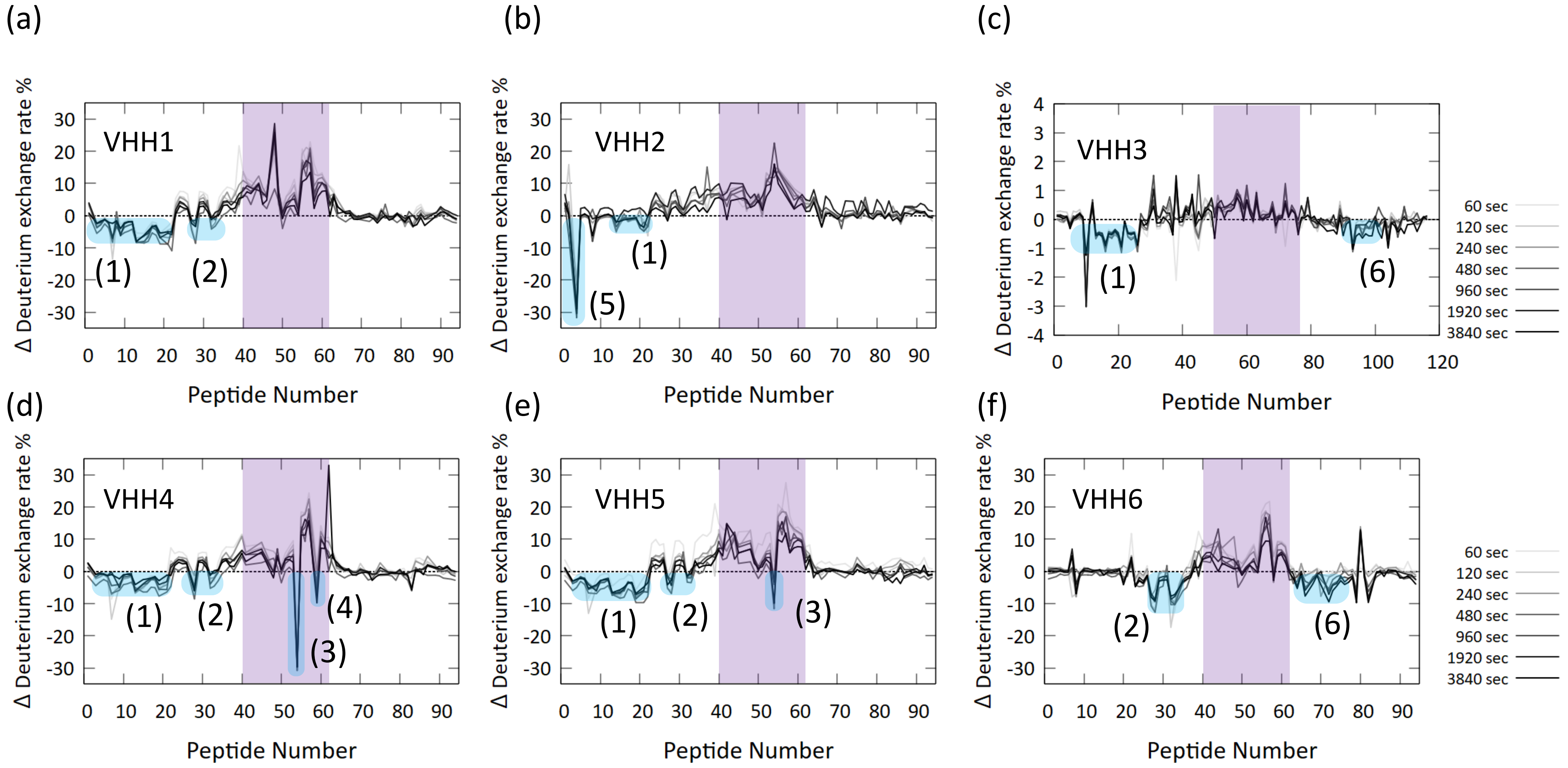

Figure S8

Epitope analysis of VHHs. (A–F) differences of HDX ratio between the data with VHHs and that without VHHs. The data including the peptides of (1) 19 to 37, (2) 38 to 52, (3) 64 to 72, (4) 80 to 89, (5) 14 to 19, and (6) 95 to 109 are shaded in light green. Data including the peptide in the grafted region derived from Glut1 are shaded in purple.

(g)

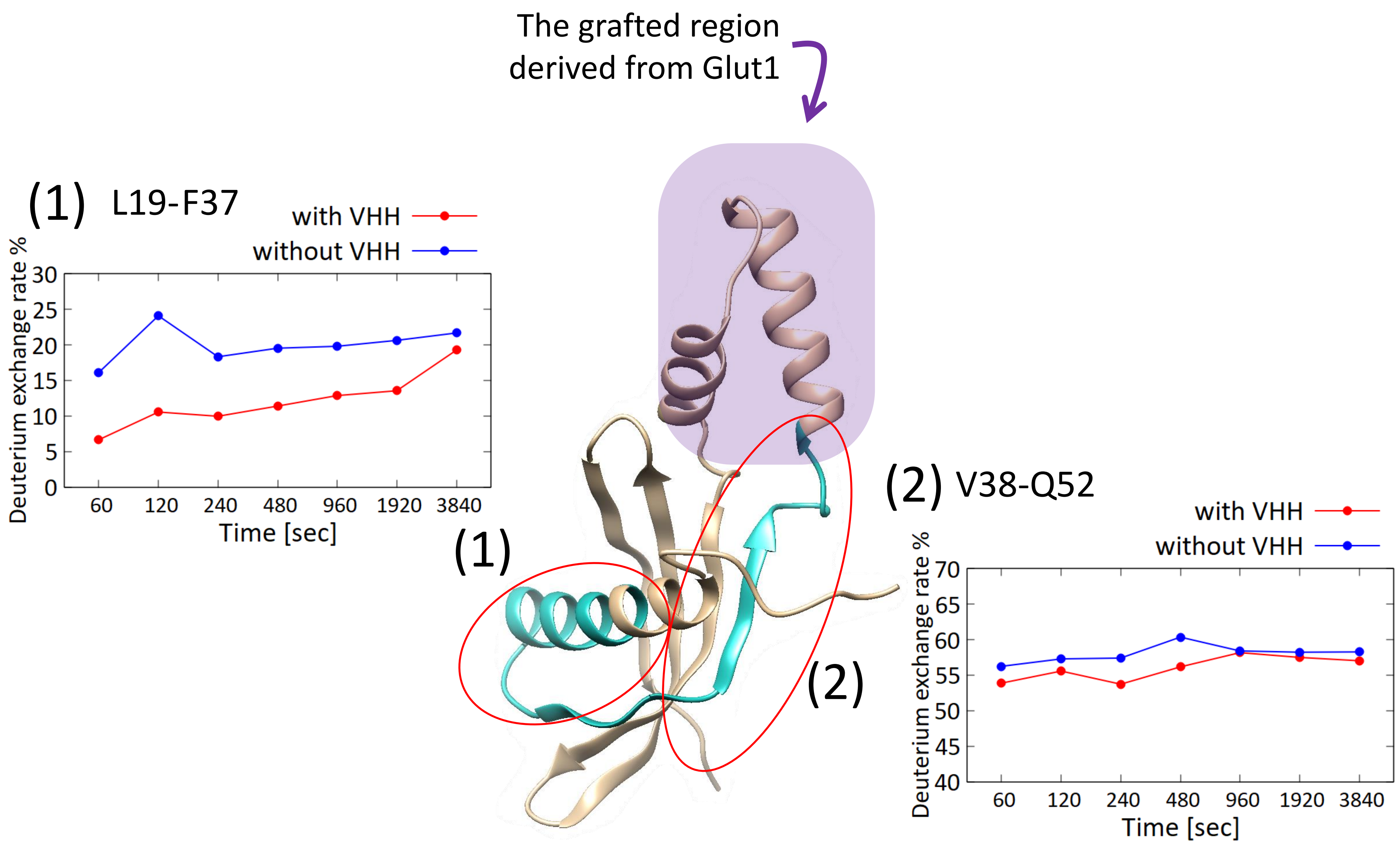

Figure S8

Epitope analysis of VHHs. (G–K) epitope analysis of (G) VHH1, (H) VHH2, (I) VHH3, (J) VHH5, and (K) VHH6. HDX ratio of representative peptides (1) to (6) as a function of time. The region containing these peptides is colored in cyan in the putative model structure of Adhiron-Glut1(35–69). The grafted region in the model structure is shaded in purple.

(h)

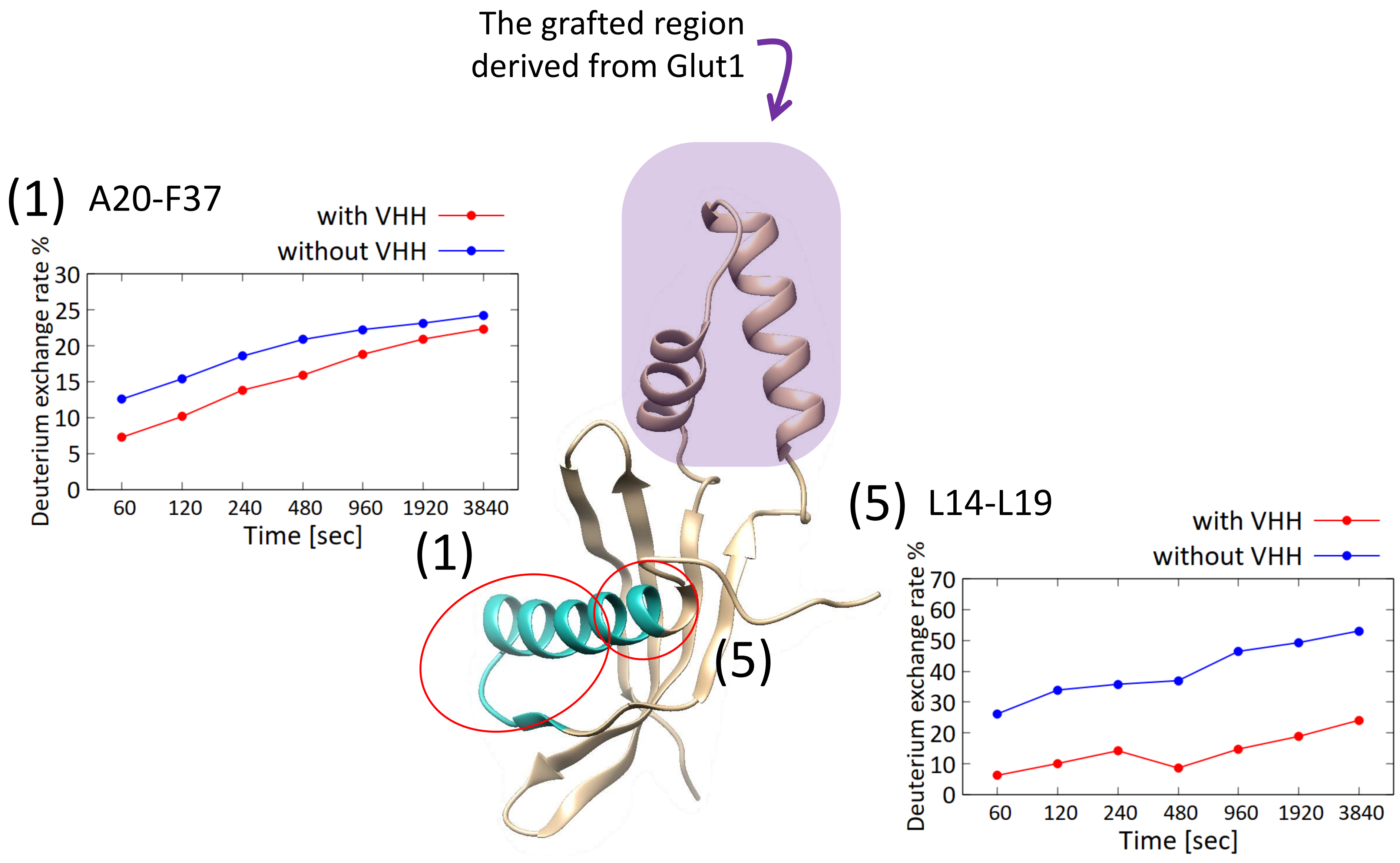

Figure S8

Epitope analysis of VHHs. (G–K) epitope analysis of (G) VHH1, (H) VHH2, (I) VHH3, (J) VHH5, and (K) VHH6. HDX ratio of representative peptides (1) to (6) as a function of time. The region containing these peptides is colored in cyan in the putative model structure of Adhiron-Glut1(35–69). The grafted region in the model structure is shaded in purple.

(i)

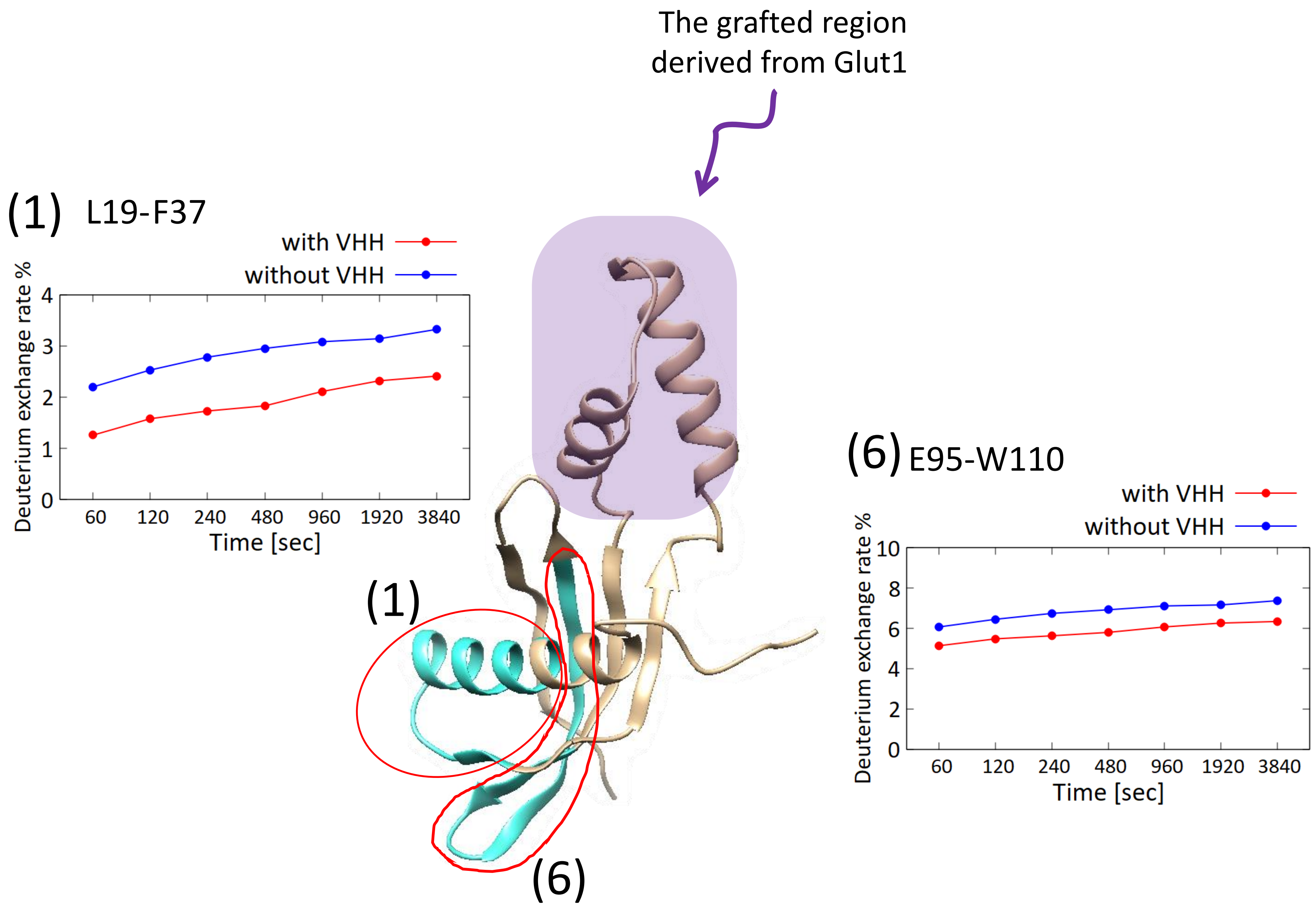

Figure S8

Epitope analysis of VHHs. (G–K) epitope analysis of (G) VHH1, (H) VHH2, (I) VHH3, (J) VHH5, and (K) VHH6. HDX ratio of representative peptides (1) to (6) as a function of time. The region containing these peptides is colored in cyan in the putative model structure of Adhiron-Glut1(35–69). The grafted region in the model structure is shaded in purple.

(j)

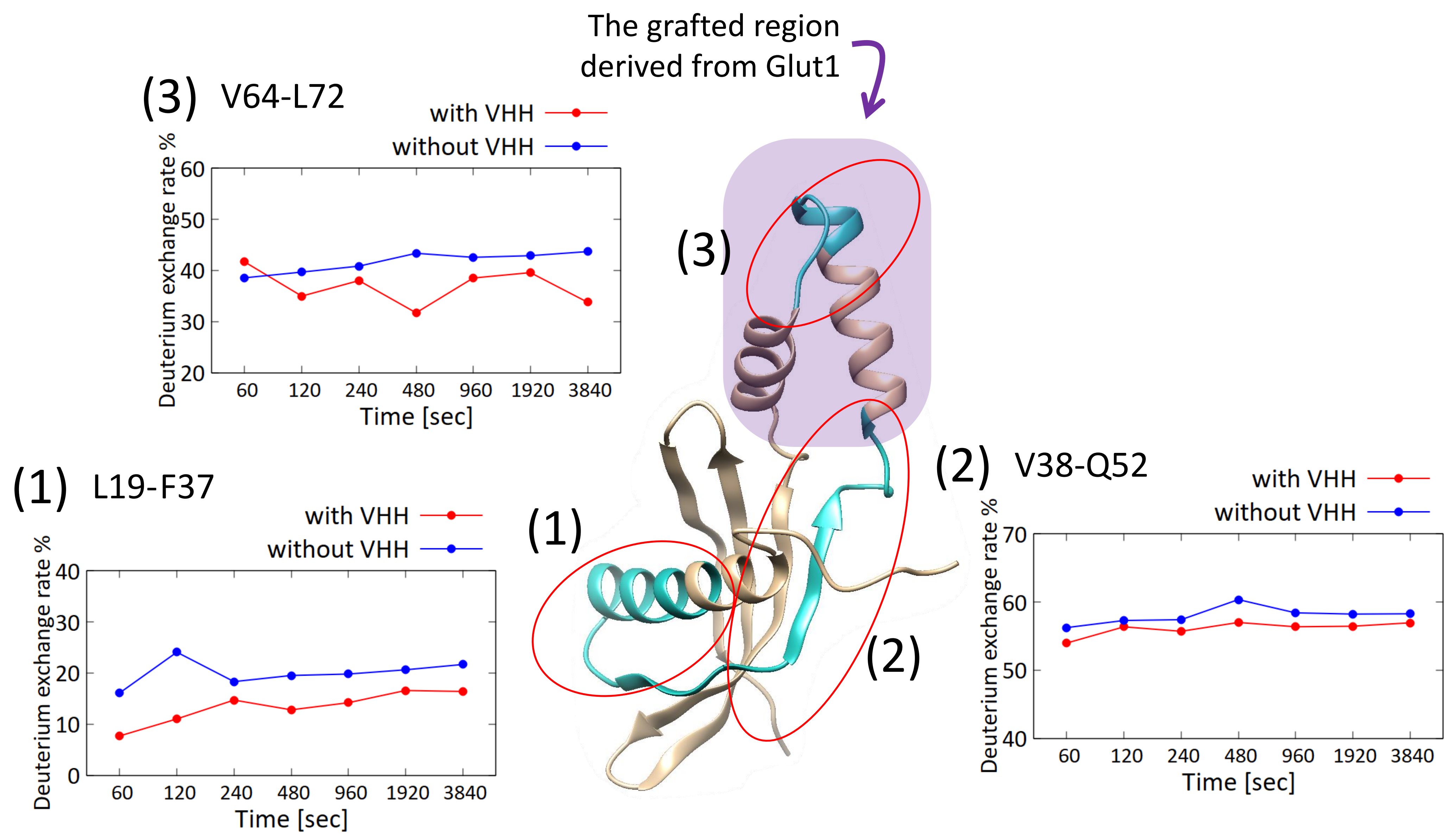

Figure S8

Epitope analysis of VHHs. (G–K) epitope analysis of (G) VHH1, (H) VHH2, (I) VHH3, (J) VHH5, and (K) VHH6. HDX ratio of representative peptides (1) to (6) as a function of time. The region containing these peptides is colored in cyan in the putative model structure of Adhiron-Glut1(35–69). The grafted region in the model structure is shaded in purple.

(k)

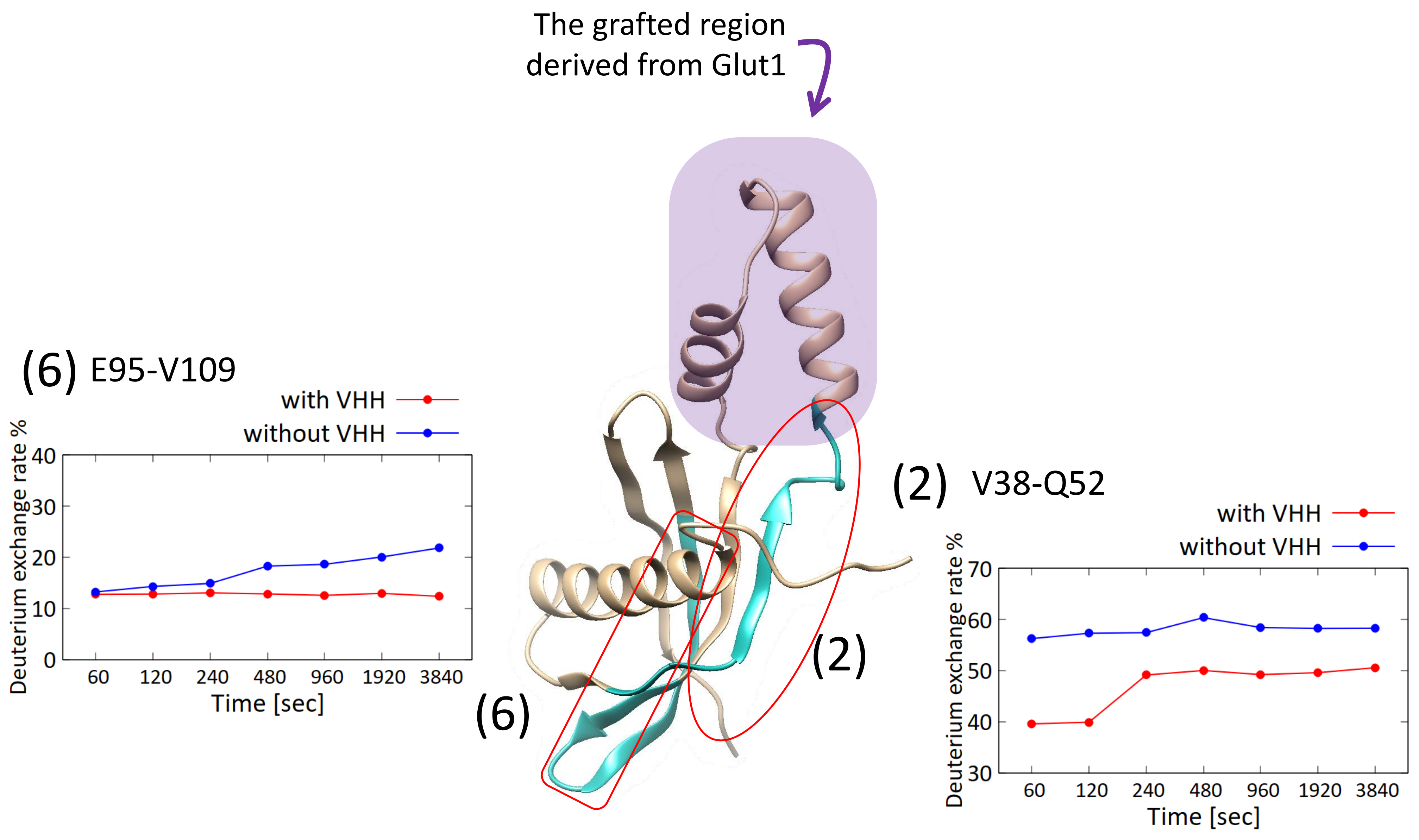

Figure S8

Epitope analysis of VHHs. (G–K) epitope analysis of (G) VHH1, (H) VHH2, (I) VHH3, (J) VHH5, and (K) VHH6. HDX ratio of representative peptides (1) to (6) as a function of time. The region containing these peptides is colored in cyan in the putative model structure of Adhiron-Glut1(35–69). The grafted region in the model structure is shaded in purple.

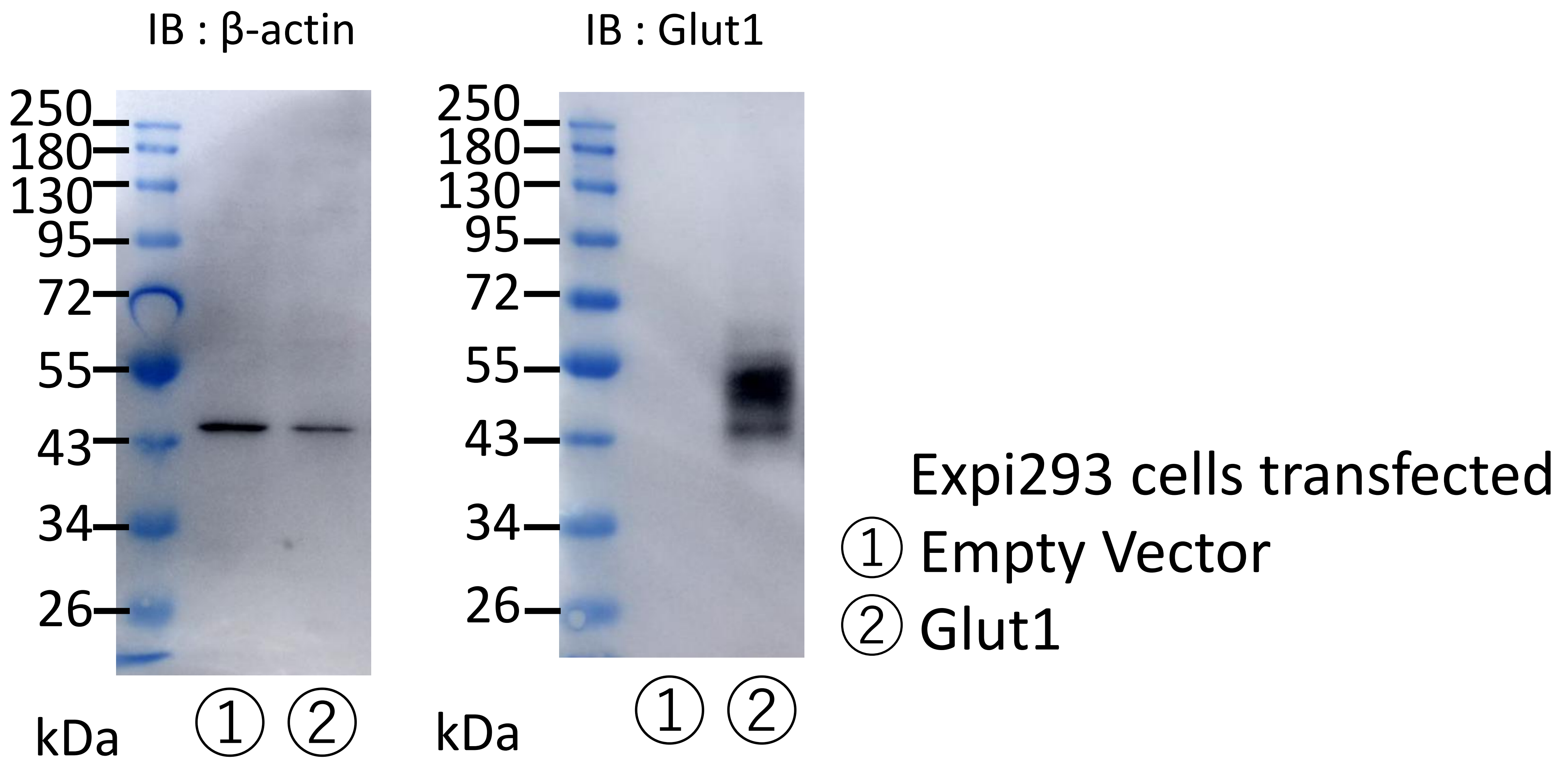

Figure S9

Confirmation of Glut1 expression. The cell lysates from Expi293 cells transfected with Glut1 or empty vector were separated by SDS-PAGE, blotted to membranes, and stained with anti-Glut1 or anti β-actin antibodies.

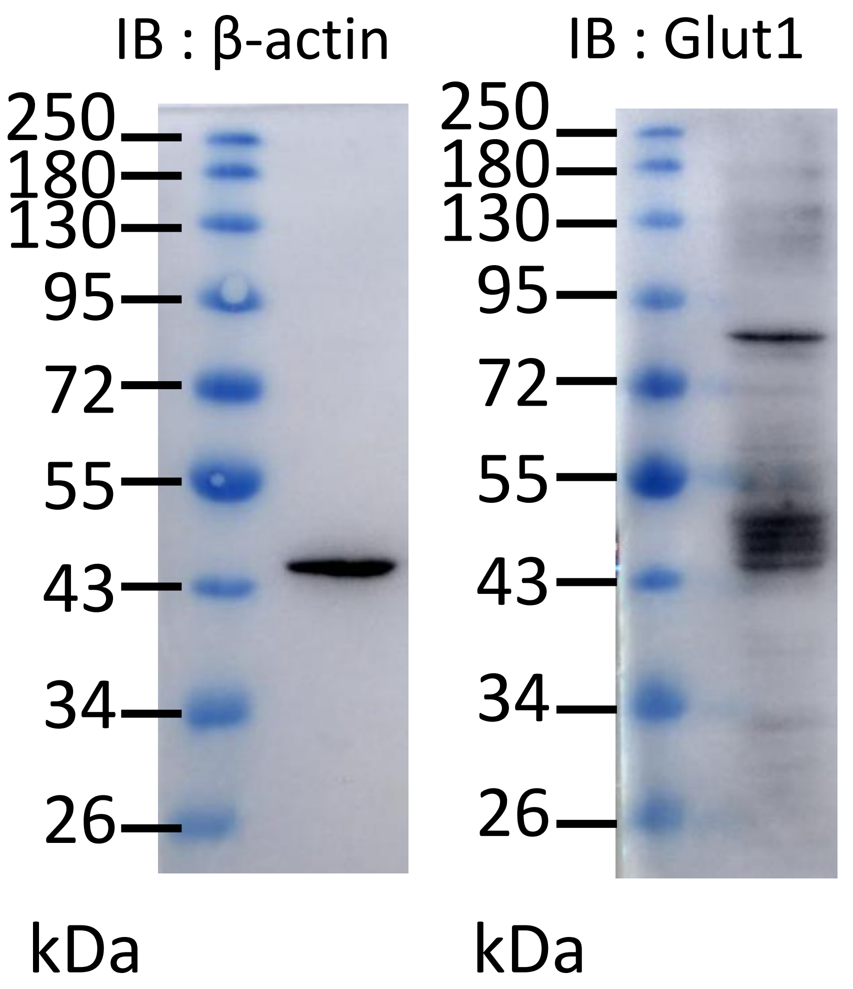

Figure S10
Confirmation of Glut1 expression. The cell lysates from HCT116 cells were separated by SDS-PAGE, blotted to membranes, and stained with anti-Glut1 or anti β-actin antibodies.

Table S1 Amino acid sequences of the obtained VHHs

	Framework1	CDR1	Framework2	CDR2
VHH1	ELQLVESGGGLVQPGGSLRLSCAAS	GFTSDRYA	IGWFRQAPGKEREGVSC	ISGHSGSK
VHH2	DVQLVESGGGLVPPGGSLRLSCEAS	GFTLDSYS	IGWFRQAPGKEREGVAC	ITRSGAST
VHH3	QLQLVESGGGLVQPGGSLTLSCAAS	GGKLDDYA	IGWFRQAPDKEREGVSC	GSSGGGSR
VHH4	EVQLVESGGGLVQPGGSLRLSCAAS	GFTLDRYA	IGWFRQAPGKEREGISC	ISSGSGSR
VHH5	ELQLVESGGGLVQPGGSLRLSCAVS	GVAFEDYA	IGWFRQAPGKEREGVSC	ISSGSGSR
VHH6	EVQLVESGGGLVQPGGSLRLSCTAS	GFTVDTYH	IGWFRQAPGKAREGVSC	ITTR-GST

	Framework3	CDR3	Framework4
VHH1	IYTDSVKGRFTISRDNVKNTVYLQMDSLKPEDTGDYYC	AAEADYYC--SGFPLYRKFGS	WGQGTQVTVSS
VHH2	NYADSVRGRFSIYRDNAKNTVYLEMNRLEPEDSAEYTC	AAQVKRDYC-TGWTYAYRMDY	WGKGTLVTVSS
VHH3	IYADSVKGRFAISRDNAKNTVYLQMNSLKPEDTAVYYC	AAEADYYC--SGFPAYRVFGS	WGQGIQVTVSS
VHH4	VYADSVKGRFTVSRDNVENTVSLEMNSLKPEDTAVYYC	AAEADYYC--SGYPIYRKFGS	WGQGTQVTVSS
VHH5	IYTDSVKGRFTVSRDNPKNTVDLQMDSLKPEDTAVYYC	AAERDYYC--SGYPTYRAFSS	WGEGTQVTVSS
VHH6	YSSESVEGRFTISRDSAKNTVYLQMNSLKPEDSAVYVC	AVDRTPGSCSVAIGASHLYDI	WGQGTQVTVSS

	Framework1	CDR1	Framework2	CDR2
Anti-Glut4 VHH	QVQLVESGGGLVQPGGSLRLTCVVS	GFTLDYYA	IGWFRQAPGTEREGVSC	IDNSGGST

	Framework3	CDR3	Framework4
Anti-Glut4 VHH	HYSASAKGRFTISRDNAKNTASLQMNSLKPEDTGIYYC	AAVSRSCDGPWPLAS	WGQGTQVTVSS

Table S2 Data summary for each HDX-MS analysis

Dataset	HDX time course (sec)	Number of peptides	Sequence coverage (%)	Average peptide length / Redundancy
The fusion protein	60, 120, 240, 480, 960, 1920, 3840	89	95.35%	16.62 / 11.47
The fusion protein / VHH1	60, 120, 240, 480, 960, 1920, 3840	85	95.35%	15.89 / 10.47
The fusion protein / VHH2	60, 120, 240, 480, 960, 1920, 3840	86	97.67%	16.35 / 10.90
The fusion protein / VHH3	60, 120, 240, 480, 960, 1920, 3840	124	97.67%	16.11 / 15.49
The fusion protein / VHH4	60, 120, 240, 480, 960, 1920, 3840	81	95.35%	15.47 / 9.71
The fusion protein / VHH5	60, 120, 240, 480, 960, 1920, 3840	82	95.35%	15.74 / 10.01
The fusion protein / VHH6	60, 120, 240, 480, 960, 1920, 3840	89	95.35%	16.65 / 11.49

Dataset	HDX time course (sec)	Number of peptides	Sequence coverage (%)	Average peptide length / Redundancy
Adhiron-Glut4	60, 120, 240, 480, 960, 1920, 3840	132	100.00%	19.89 / 19.74
Adhiron-Glut4 / anti-Glut4 VHH	60, 120, 240, 480, 960, 1920, 3840	109	100.00%	19.42 / 15.92

Table S3 Thermodynamic parameters provided by ITC measurements

n = 3	N	K_D (nM)	ΔH (kcal/mol)	-TΔS (kcal/mol)
VHH1				
Adhiron-Glut1	1.23±0.06	49.5±27.0	-16.5±0.34	5.98±1.02
Adhiron-WT	1.11±0.03	0.16±0.05	-19.3±0.03	5.92±0.18
VHH2				
Adhiron-Glut1	1.22±0.02	9.53±1.96	-13.0±0.28	2.03±0.37
Adhiron-WT	1.13±0.02	6.57±1.01	-13.4±0.22	2.27±0.32
VHH3				
Adhiron-Glut1	1.21±0.05	18.9±11.8	-10.9±0.87	-0.04±0.69
Adhiron-WT	1.11±0.00	2.70±0.52	-13.8±0.13	2.09±0.25
VHH4				
Adhiron-Glut1	1.24±0.02	9.24±4.74	-11.4±0.46	0.24±0.70
Adhiron-WT	1.07±0.02	4.71±2.37	-12.7±0.19	1.22±0.24
VHH5				
Adhiron-Glut1	1.13±0.01	7.21±1.70	-13.6±0.04	2.47±0.14
Adhiron-WT	1.01±0.01	1.39±0.29	-14.7±0.26	2.54±0.27
VHH6				
Adhiron-Glut1	1.03±0.00	2.16±0.40	-11.4±0.05	-0.46±0.16
Adhiron-WT	0.97±0.02	0.58±0.34	-11.1±0.10	-1.77±0.34

n = 3	N	K_D (nM)	ΔH (kcal/mol)	-TΔS (kcal/mol)
Anti-Glut4 VHH				
Adhiron-Glut4	1.02±0.08	3.67±1.15	-24.9±0.48	13.2±0.40
Adhiron-WT	--	--	--	--

Data are mean ± standard error of the mean (s.e.m.)

Table S4 Ratio and the number of residues for each secondary structure type in Adhiron-WT and the fusion proteins analyzed by the BeStSel program

	Adhiron-WT	Adhiron-Glut1 (35-54)	Adhiron-Glut1 (35-69)	Adhiron-Glut1 (35-69) mutant
Number of residues	92	118	129	129
Helix:	22.5[a] / 20.7[b]	16.3 / 19.2	20.4 / 26.3	18.2 / 23.5
Antiparallel:	17.2 / 15.8	29.5 / 34.8	22.8 / 29.4	30.1 / 38.8
Parallel:	12.9 / 11.9	7.9 / 9.3	10.4 / 13.4	9.3 / 12.0
Turn:	12.0 / 11.0	11.0 / 13.0	10.1 / 13.0	10.6 / 13.7
Others:	35.4 / 32.6	35.4 / 41.8	36.2 / 46.7	31.7 / 40.9

* a: The ratio of each secondary structure type [%]
b: The number of residues of secondary structure type [residues]
